# Supplementary material for: Model certainty in cellular network-driven processes with missing data
Source: PLoS Comput Biol. 2023 Apr 26;19(4):e1011004. doi: 10.1371/journal.pcbi.1011004 (PMC10166548; doi:10.1371/journal.pcbi.1011004)
Supplement: S1 Text — The supporting information provides method details. Tables A-E describe parameters and reactions included in the aEARM. The following figures describe the prior and posterior distributions of aEARM parameters calibrated to using different dataset and measurement models. Also included (Figs A, G, K and R) are various posterior predictions for aEARM variables. This file contains a table of contents for further details. (DOCX) [file pcbi.1011004.s001.docx]

**Supplemental Methods**

**Table of Contents**

**Table A – Initial Conditions………………………………………………………………………………………………………………….2**

**Table B – Reactions for unrelated signaling……………………………………………..………………………………………….2**

**Table C – Parameterizations of Ordinal Measurement Model………………..……………………………………………3**

**Table D – Ground Truth Parameterization of Nominal Measurement Model.………………………………………4**

**Table E – Ground Truth Parameterization of aEARM……………………………..……………………………………………4**

**Table F – Gelman Rubin Values of each model calibration…..**(see <https://doi.org/10.5281/zenodo.7007655>)

**Fig A – PARP cleavage dynamics of aEARM trained to Flourescence EC-RP data...............................5**

**Fig B – Model parameters calibrated to a Fluorescence Dataset......................................................7**

**Fig C – Model parameters calibrated to an Oridinal Dataset............................................................9**

**Fig D – Model parameters calibrated to an Ordinal Dataset...........................................................11**

**Fig E – Model parameters calibrated to an Ordinal Dataset............................................................13**

**Fig F – Model parameters calibrated to an Ordinal Dataset............................................................15**

**Fig G – Predicted Initiator caspase and DISC colocalization dynamics of aEARM trained to ordinal and mixed ordinal/nominal datasets..................................................................................................17**

**Fig H – Model parameters calibrated to a Nominal Cell Death Dataset...........................................18**

**Fig I – Model parameters calibrated to Ordinal IC-DISC Dataset....................................................20**

**Fig J – Model parameters calibrated to Mixed Ordinal and Nominal Dataset...............................22**

**Fig K – Predicted PARP cleavage dynamics of aEARM trained to ordinal data using different measurement model priors.................................................................................................................24**

**Fig L – Predicted PARP cleavage dynamics of aEARM trained to ordinal data using different ad hoc parameterizations of the measurement model...................................................................................25**

**Fig M – Model parameters calibrated to Ordinal Dataset using Uniform priors on Measurement Model Parameters..............................................................................................................................26**

**Fig N - Model parameters calibrated to Ordinal Dataset using Cauchy priors on Measurement Model Parameters..............................................................................................................................28**

**Fig O - Model parameters calibrated to Ordinal Dataset using Cauchy priors on Measurement Model Parameters..............................................................................................................................30**

**Fig P - Model parameters calibrated to Ordinal Dataset using Fixed ad hoc Measurement Model Parameters.........................................................................................................................................32**

**Fig Q - Model parameters calibrated to Ordinal Dataset using Fixed ad hoc Measurement Model Parameters.........................................................................................................................................34**

**Fig R - Predicted Bid truncation dynamics of aEARM trained to published fractional cell death data....................................................................................................................................................36**

**Fig S - Model parameters calibrated to published fractional cell death data.................................37**

**Fig T - Reprinted from Roux et al. [1] caspase activity as a predictor of apoptosis in HeLa cells....................................................................................................................................................39**

**Supplemental Methods Details**

Table A - Initial conditions:

| **Species** | **Symbol** | **Value** | **Notes** |
| --- | --- | --- | --- |
| Ligand (TRAIL) | ‘L_0’ | 3000 | 3000 copies per cell equates to 50ng/mL |
| Receptor | ‘R_0’ | 200 |  |
| DISC | ‘DISC_0’ | 0 | The death inducing signaling complex (DISC) is initially absent |
| Initiator Caspase | ‘IC_0’ | 2.0e4 | Caspase 8 |
| Effector Caspase | ‘EC_0’ | 1.0e4 | Caspase 3 |
| MOMP signal* | ‘MOMP_sig_0’ | 1.0e5 | Uses the EARM initial value for Smac |
| PARP | ‘PARP_0’ | 1.0e6 | PARP (Caspase 3 substrate and apoptosis marker) |
| Three Unrelated* Signaling Molecules | ‘USM1_0’ | 1.0e3 | These signaling molecules do not interact with aEARM and are linked together via a separate set of activation and inactivation reactions (Supplemental table 2) |
|  | ‘USM2_0’ | 1.0e3 |  |
|  | ‘USM3_0’ | 1.0e3 |  |

^*^indicates values that were subjected log-normal extrinsic noise. Additionally, rate parameter ‘kc0’ (which catalyzes the formation of DISC) was subjected to extrinsic noise using the same procedure as for those noted in the table. All species uses copies per cell as their units.

Table B – Reactions for unrelated signaling molecules

| USM1 catalyzes USM2 activation | ${USM1}^{*}+USM2 \rightleftharpoons{USM1}^{*}:USM2 \longrightarrow{USM1}^{*}+{USM2}^{*}$ |  |
| --- | --- | --- |
| USM2 catalyzes USM3 activation | ${USM2}^{*}+USM3 \rightleftharpoons{USM2}^{*}:USM3 \longrightarrow{USM2}^{*}+{USM3}^{*}$ |  |
| USM3 catalyzes USM1 activation | ${USM3}^{*}+USM1 \rightleftharpoons{USM3}^{*}:USM1 \longrightarrow{USM3}^{*}+{USM1}^{*}$ |  |
| USM1 catalyzes USM3 inactivation | ${USM1}^{*}+ {USM3}^{*} \rightleftharpoons{USM1}^{*}:{USM3}^{*} \longrightarrow{USM1}^{*}+USM3$ |  |
| USM2 catalyzes USM1 inactivation | ${USM2}^{*}+ {USM1}^{*} \rightleftharpoons{USM2}^{*}:{USM1}^{*} \longrightarrow{USM2}^{*}+USM1$ |  |
| USM3 catalyzes USM2 inactivation | ${USM3}^{*}+ {USM2}^{*} \rightleftharpoons{USM3}^{*}:{USM2}^{*} \longrightarrow{USM3}^{*}+USM2$ |  |

^*^ indicates activated.

Monotonically increasing values would correlate highly with TRAIL dependent species in aEARM, as an artifact of the aEARM species all having nonequilibrium values at t=0. This activation and inactivation scheme can produce slow oscillations, which decrease their correlation with TRAIL dependent species in aEARM.

Table C – Parameterizations of Ordinal Measurement Model

| “Ground Truth Parameterization” | $\alpha_{tBID}=50.0$, $\boldsymbol{\beta}_{tBID}=(0.03, 0.40, 0.82, 0.97)$  $\theta_{tBID}=(0.03, 0.37, 0.42, 0.15)$  $\alpha_{cPARP}=50.0$, $\boldsymbol{\beta}_{cPARP}=(0.03, 0.20, 0.97)$  $\theta_{cPARP}=(0.03, 0.17, 0.77)$  $\alpha_{IC-DISC}=50.0$, $\boldsymbol{\beta}_{IC\_DISC}=(0.05, 0.40, 0.85)$  $\theta_{IC-DISC}=(0.05, 0.35, 0.45)$ |
| --- | --- |
| *Ad hoc* parameterization (Case 1) | $\alpha_{tBID}=50.0$, $\boldsymbol{\beta}_{tBID}=(0.00, 0.33, 0.67, 1.00)$  $\theta_{tBID}=(0.00, 0.33, 0.34, 0.33)$  $\alpha_{cPARP}=50.0$, $\boldsymbol{\beta}_{cPARP}=(0.00, 0.50, 1.00)$  $\theta_{cPARP}=(0.00, 0.50, 0.50)$ |
| *Ad hoc* parameterization (Case 2) | $\alpha_{tBID}=50.0$, $\boldsymbol{\beta}_{tBID}=(0.20, 0.40, 0.60, 0.80)$  $\theta_{tBID}=(0.20, 0.20, 0.20, 0.20)$  $\alpha_{cPARP}=50.0$, $\boldsymbol{\beta}_{cPARP}=(0.25, 0.50, 0.75)$  $\theta_{CPARP}=(0.25, 0.25, 0.25)$ |
|  |  |

We express each offset, $\beta_{j}$, using a distance, $\theta_{j}$, from its preceding offset term; $\beta_{j}= \beta_{j-1}+ \theta_{j}$. The first offset, $\beta_{0}= \theta_{0}$, and subsequent distance, $\theta_{j}$. Figs C-F, I, J, and M-Q contain prior and posterior plots of $\theta_{j}$. For example: “coefficients__tBID_blot__theta_1” corresponds to $\theta_{tBID, 1}$ (the first offset distance $\theta_{1}$ for tBID).

Table D – Ground Truth Parameterization of Nominal Measurement Model

| “Ground Truth Parameterization” | $\alpha=-4.0$ (slope term)  $\beta=0.25$ (intercept term) $\beta_{unrelated signal}=0.0$  $\beta_{\max tBID rate}=-0.25$  $\beta_{time at\max tBID rate}=1.0$ |
| --- | --- |
|  |  |

Table E – Ground Truth Parameterization of aEARM

| kf0 | 1.15594933e-07 | kr2 | 6.87631084e-02 | kc4 | 3.39059104e-03 | kf7 | 1.98195485e-05 |
| --- | --- | --- | --- | --- | --- | --- | --- |
| kr0 | 1.04056134e-05 | kc2 | 1.09271827e-02 | kf5 | 8.69183082e-10 | kr7 | 1.82182416e-04 |
| kc0 | 1.12028665e-05 | kf3 | 3.82096809e-06 | kr5 | 1.15619903e-03 | kc7 | 3.90039620e-03 |
| kf1 | 1.93083645e-06 | kr3 | 1.39230000e-05 | kc5 | 8.49612026e-06 | kf8 | 1.76040074e-05 |
| kr1 | 1.71446702e-03 | kc3 | 2.95330594e-02 | kf6 | 7.69174202e-07 | kr8 | 1.34854804e-03 |
| kc1 | 3.29355599e-01 | kf4 | 1.61609297e-05 | kr6 | 7.35417590e-04 | kc8 | 1.67799299e-01 |
| kf2 | 1.48867282e-08 | kr4 | 1.71818138e-02 | kc6 | 5.18485726e-03 | kc9 | 8.28571278e-08 |

All $kf$ use $cell/(copies\times s)$ as their units. All $kr$ and $kc$ use 1/s as their units.

Table F – Gelman Rubin Values for each model calibration

see <https://doi.org/10.5281/zenodo.7007655>

**
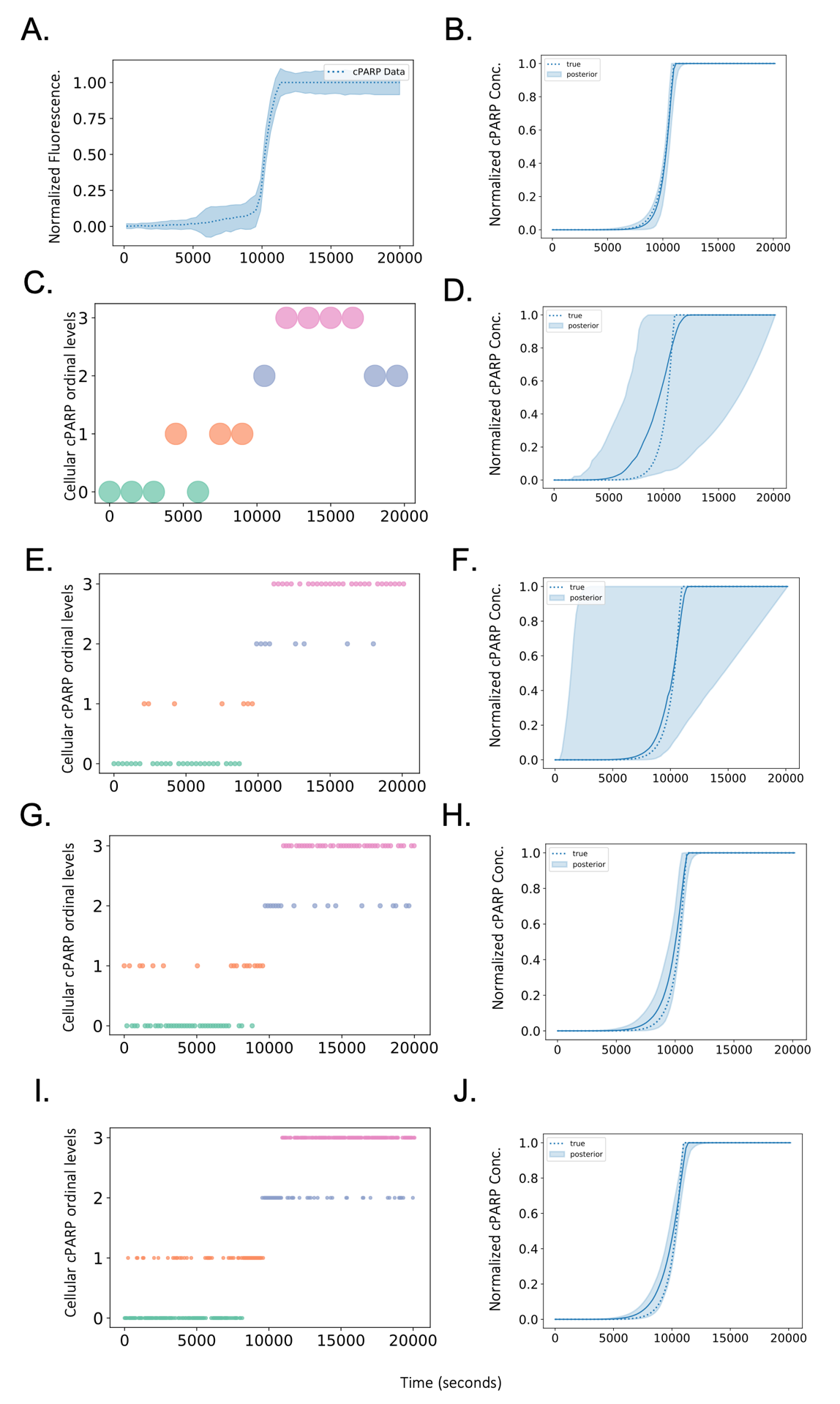
**

**Fig A (Also see Fig 1A): PARP cleavage dynamics of aEARM trained to Fluorescence EC-RP data.** Bayesian optimization of aEARM parameters trained to Effector caspase reporter (EC-RP) fluorescence time-course measurements at 180s intervals (i.e., a proxy for PARP cleavage) shown in the top row (left) and IC-RP fluorescence time-course (See Fig 1B) data from Albeck et al [21]).The plot shows the mean (dotted line) +/- 1 standard deviation (shaded region) for each time point. In the top row, right, the 95% credible region of posterior predictions (shaded region) for cPARP concentration in aEARM, calibrated to fluorescence measurements of IC-RP and EC-RP data. The median prediction (solid-line) and true (dotted line) cPARP concentration trajectories are shown. Left column second through fifth rows show ordinal measurements of cPARP at occurring at every 1500 (second row), 300, 180 and 60 (bottom row) second timepoint. The 95% credible region of posterior predictions of cPARP dynamics for aEARM calibrated to each corresponding ordinal dataset is show in each row (i.e., same row) on the right. I.e., the 95% credible region of predictions (shaded region), median prediction (solid line) and true (dotted line) for cPARP dynamics. The corresponding plots for tBID ordinal measurements and predictions are found in Fig 1.

**Fig B (part 1) (Also, see Figure 1B top row): Model parameters calibrated to a Fluorescence Dataset** Parameters for aEARM were calibrated to fluorescence time-course measurements of EC-RP and IC-RP at every 180s interval. Prior (blue) and posterior (orange) distributions log_10_ of the value of parameter are shown.

**Fig B (part 2) (Also, see Fig 1B top row): Model parameters (remaining) calibrated to a Fluorescence Dataset** Parameters for aEARM were calibrated to fluorescence time-course measurements of EC-RP and IC-RP at every 180s interval. Prior (blue) and posterior (orange) distributions log_10_ of the value of parameter are shown.

**Fig C (Also, see Fig 1B bottom row): Model parameters calibrated to an Ordinal Dataset** Parameters for aEARM were calibrated to ordinal values of tBID and cPARP abundance at every 60s interval. Prior (blue) and posterior (orange) distributions log_10_ of the value of parameter are shown.

**Fig C (part 2) (Also, see Fig 1B, bottom row): Model parameters calibrated to an Ordinal Dataset** Parameters for aEARM were calibrated to ordinal values of tBID and cPARP abundance at every 60s interval. Prior (blue) and posterior (orange) distributions log_10_ of the value of the aEARM parameter are shown. Prior and posterior distributions of the value of measurement model coefficients are also shown (these are not log-scale).

**Fig D (Also, see Fig 1B, 4^th^ row): Model parameters calibrated to an Ordinal Dataset** Parameters for aEARM were calibrated to ordinal values of tBID and cPARP abundance at every 180s interval. Prior (blue) and posterior (orange) distributions log_10_ of the value of parameter are shown.

**Fig D (part 2) (Also, see Fig 1B, 4^th^ row): Model parameters calibrated to an Ordinal Dataset** Parameters for aEARM were calibrated to ordinal values of tBID and cPARP abundance at every 180s interval. Prior (blue) and posterior (orange) distributions log_10_ of the value of the aEARM parameter are shown. Prior and posterior distributions of the value of measurement model coefficients are also shown (these are not log-scale).

**Fig E (Also, see Fig 1B, 3^rd^ row): Model parameters calibrated to an Ordinal Dataset** Parameters for aEARM were calibrated to ordinal values of tBID and cPARP abundance at every 300s interval. Prior (blue) and posterior (orange) distributions log_10_ of the value of parameter are shown.

**Fig E (part 2) (Also see Fig 1B, 3^rd^ row): Model parameters calibrated to an Ordinal Dataset** Parameters for aEARM were calibrated to ordinal values of tBID and cPARP abundance at every 300s interval. Prior (blue) and posterior (orange) distributions log_10_ of the value of the aEARM parameter are shown. Prior and posterior distributions of the value of measurement model coefficients are also shown (these are not log-scale).

**Fig F (Also, see Fig 1B, 2^nd^ row): Model parameters calibrated to an Ordinal Dataset** Parameters for aEARM were calibrated to ordinal values of tBID and cPARP abundance at every 1500s interval. Prior (blue) and posterior (orange) distributions log_10_ of the value of parameter are shown.

**Fig F (part 2) (Also, see Fig 1B, 2^nd^ row): Model parameters calibrated to an Ordinal Dataset** Parameters for aEARM were calibrated to ordinal values of tBID and cPARP abundance at every 1500s interval. Prior (blue) and posterior (orange) distributions log_10_ of the value of the aEARM parameter are shown. Prior and posterior distributions of the value of measurement model coefficients are also shown (these are not log-scale).


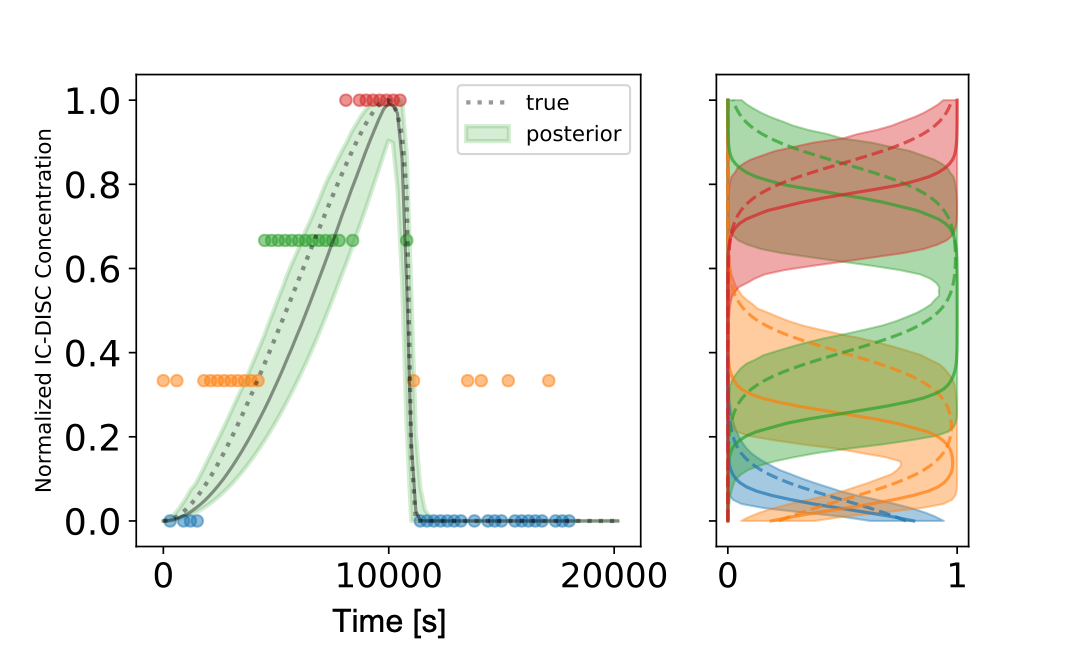


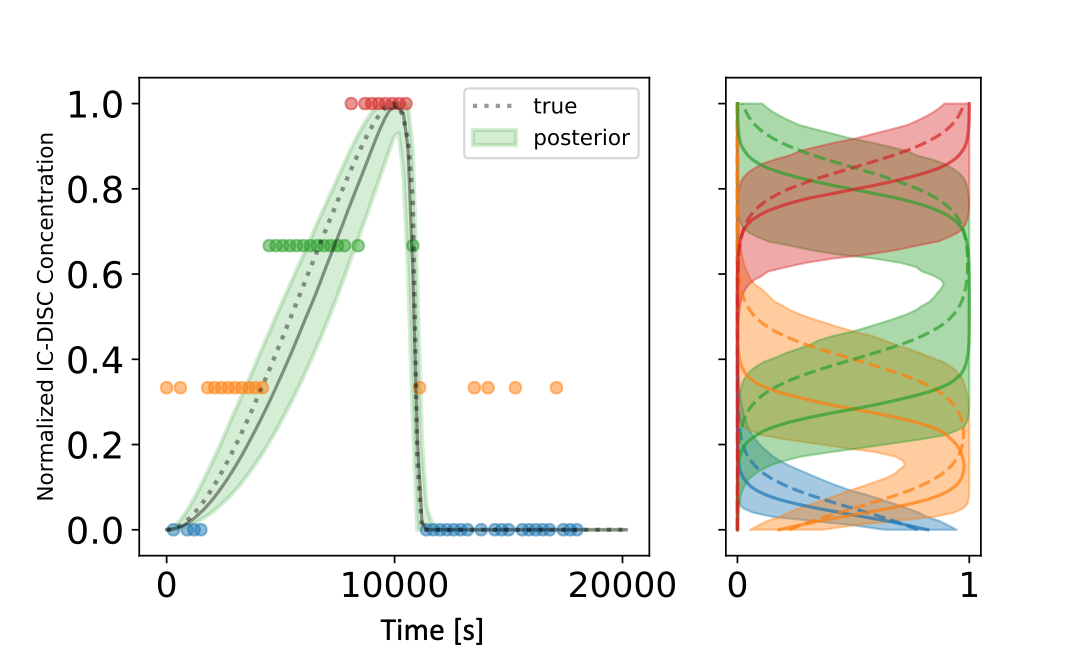


**Fig G. Predicted Initiator caspase and DISC colocalization dynamics of aEARM trained to ordinal and mixed ordinal/nominal datasets.**

Top: The 95% credible region (shaded region) of posterior predictions of IC-DISC dynamics of aEARM calibrated to ordinal IC-DISC data (shown in Fig 2B).

Bottom: The 95% credible region (shaded region) of posterior predictions of IC-DISC dynamics of aEARM calibrated to a mixed dataset containing the nominal data (shown in Fig 2) the ordinal IC-DISC data (shown in Fig 2B).

The median predictions (solid-line) and true (dotted line) are also plotted. The adjacent panels give the 95% credible region of posterior predictions (shaded regions) for the probability of class membership (x-axis) as a function of aEARM-simulated normalized IC-DISC concentration (y-axis). The ordinal categories are color coded and plotted in ascending order.

**Fig H (Also, see Fig 4, left column): Model parameters calibrated to a Nominal Cell Death Dataset** Parameters for aEARM were calibrated to nominal observations of cell death vs survival. Prior (blue) and posterior (orange) distributions log_10_ of the value of parameter are shown.

**Fig H (part 2) (Also,see Fig 4, left column): Model parameters calibrated to a Cell Dataset** Parameters for aEARM were calibrated to nominal observations of cell death vs survival. Prior (blue) and posterior (orange) distributions log_10_ of the value of the aEARM parameter are shown. Prior and posterior distributions of the value of measurement model coefficients: slope ($\alpha$ in S. Table 4), intercept ($\beta$), unrelated signal coef ($\beta_{unrelated signal}$), tBID obs coef ($\beta_{\max tBID rate}$) and time coef ($\beta_{time at\max tBID rate}$) are also shown (these are not log-scale).

**Fig I (Also, see Fig 2B): Model parameters calibrated to Ordinal IC-DISC Dataset** Parameters for aEARM were calibrated to ordinal values of IC-DISC abundance at every 300s interval. Prior (blue) and posterior (orange) distributions log_10_ of the value of parameter are shown.

IC_DISC coeff

IC_DISC theta 1

IC_DISC theta 2

IC_DISC theta 3

**Fig I (part 2) (Also, see Fig 2B): Model parameters calibrated to Ordinal IC-DISC Dataset** Parameters for aEARM were calibrated to ordinal values of IC-DISC abundance at every 300s interval. Prior (blue) and posterior (orange) distributions log_10_ of the value of parameter are shown. Prior and posterior distributions of the value of measurement model coefficients are also shown (these are not log-scale).

**Fig J (Also, see Figs 2C and 4, right column): Model parameters calibrated to Mixed Ordinal and Nominal Dataset** Parameters for aEARM were calibrated to ordinal values of IC-DISC abundance at every 300s interval and to nominal observations of cell death vs survival. Prior (blue) and posterior (orange) distributions log_10_ of the value of parameter are shown.


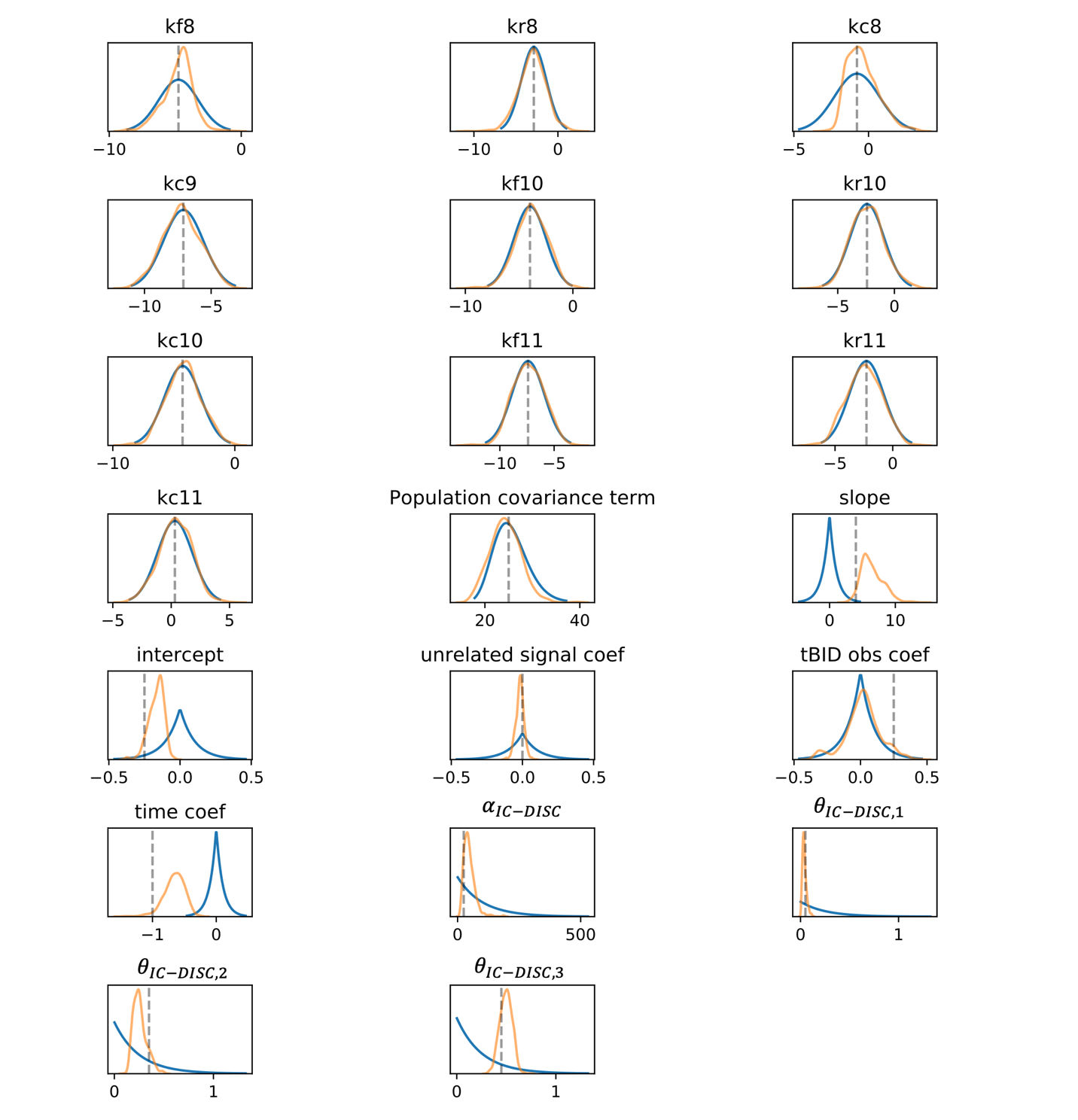

**Fig J (part 2) (Also, see Figs 2C and 4, right column): Model parameters calibrated to Mixed Ordinal and Nominal Dataset** Parameters for aEARM were calibrated to ordinal values of IC-DISC abundance at every 300s interval and to nominal observations of cell death vs survival. Prior (blue) and posterior (orange) distributions log_10_ of the value of parameter are shown. Prior and posterior distributions of the value of measurement model coefficients: For the nominal dataset: slope ($\alpha$ in S. Table 4), intercept ($\beta$), unrelated signal coef ($\beta_{unrelated signal}$), tBID obs coef ($\beta_{\max tBID rate}$) and time coef ($\beta_{time at\max tBID rate}$) and the ordinal IC_DISC dataset are also shown (these are not log-scale).

**Fig K (Also, see Fig3C, 3D and 3E): Predicted PARP cleavage dynamics of aEARM trained to ordinal data using different measurement model priors.**

The 95% credible region of posterior predictions (shaded region) of cPARP dynamics of aEARM calibrated to ordinal values of tBID and cPARP at every 60s interval. Uniform, Cauchy (scale=0.05) and Cauchy (scale=0.005) prior distributions for the parameterizations for the measurement model, respectively. In each, the median prediction (solid line) and true (dotted line) cPARP dynamic sare also shown. The adjacent panels give the 95% credible region of posterior predictions of the probability of class membership (x-axis) as a function of normalized cPARP concentration (y-axis).

**Fig L (Also, see Fig 3A and 3B): Predicted PARP cleavage dynamics of aEARM trained to ordinal data using different *ad hoc* parameterizations of the measurement model.** The 95% credible region of posterior predictions (shaded region) of cPARP dynamics for aEARM calibrated to ordinal measurements of tBID and cPARP at every 60s interval. Two fixed parameterizations for the measurement model. The adjacent panels plot the measurement models predicted probability of class membership (x-axis) as a function of normalized cPARP concentration (y-axis). The adjacent panels give the 95% credible region of posterior predictions of the probability of class membership (x-axis) as a function of normalized cPARP concentration (y-axis).

**Fig M (Also, see Fig 3C): Model parameters calibrated to Ordinal Dataset using Uniform priors on Measurement Model Parameters** Parameters for aEARM were calibrated to ordinal values of tBID and cPARP abundance at every 60s interval. Prior (blue) and posterior (orange) distributions log_10_ of the value of parameter are shown. Uniform priors were placed on the measurement model parameters (these are not log-scale).

**Fig M (part 2) (Also, see Fig 3C): Model parameters calibrated to Ordinal Dataset using Uniform priors on Measurement Model Parameters** Parameters for aEARM were calibrated to ordinal values of tBID and cPARP abundance at every 60s interval. Prior (blue) and posterior (orange) distributions log_10_ of the value of parameter are shown. Uniform priors were placed on the measurement model parameters (these are not log-scale).

**Fig N (Also, see Fig 3D): Model parameters calibrated to Ordinal Dataset using Cauchy priors on Measurement Model Parameters.** Parameters for aEARM were calibrated to ordinal values of tBID and cPARP abundance at every 60s interval. Prior (blue) and posterior (orange) distributions log_10_ of the value of parameter are shown. Cauchy priors with a scale term of 0.05 were placed on the measurement model parameters (these are not log-scale).

**Fig N (part 2) (Also, see Fig 3D): Model parameters calibrated to Ordinal Dataset using Cauchy priors on Measurement Model Parameters.** Parameters for aEARM were calibrated to ordinal values of tBID and cPARP abundance at every 60s interval. Prior (blue) and posterior (orange) distributions log_10_ of the value of parameter are shown. Cauchy priors with a scale term of 0.05 were placed on the measurement model parameters (these are not log-scale)

**Fig O (Also, see Fig 3E): Model parameters calibrated to Ordinal Dataset using Cauchy priors on Measurement Model Parameters.** Parameters for aEARM were calibrated to ordinal values of tBID and cPARP abundance at every 60s interval. Prior (blue) and posterior (orange) distributions log_10_ of the value of parameter are shown. Cauchy priors with a scale term of 0.005 were placed on the measurement model parameters (these are not log-scale).

**Fig O (part 2) (Also, see Fig 3E): Model parameters calibrated to Ordinal Dataset using Cauchy priors on Measurement Model Parameters.** Parameters for aEARM were calibrated to ordinal values of tBID and cPARP abundance at every 60s interval. Prior (blue) and posterior (orange) distributions log_10_ of the value of parameter are shown. Cauchy priors with a scale term of 0.005 were placed on the measurement model parameters (these are not log-scale).

**Fig P (Also, see Fig 3A and 3B): Model parameters calibrated to Ordinal Dataset using Fixed *ad hoc* Measurement Model Parameters.** Parameters for aEARM were calibrated to ordinal values of tBID and cPARP abundance at every 60s interval. Prior (blue) and posterior (orange) distributions log_10_ of the value of parameter are shown. Fixed *ad hoc* values were applied to the measurement model parameters (see Supplemental Table C (Case 1))

**Fig P (part 2) (Also, see Fig 3A and 3B): Model parameters calibrated to Ordinal Dataset using Fixed *ad hoc* Measurement Model Parameters.** Parameters for aEARM were calibrated to ordinal values of tBID and cPARP abundance at every 60s interval. Prior (blue) and posterior (orange) distributions log_10_ of the value of parameter are shown. Fixed *ad hoc* values were applied to the measurement model parameters (see Supplemental Table C (Case 1))

**Fig Q (Also, see Fig 3A and 3B): Model parameters calibrated to Ordinal Dataset using Fixed *ad hoc* Measurement Model Parameters.** Parameters for aEARM were calibrated to ordinal values of tBID and cPARP abundance at every 60s interval. Prior (blue) and posterior (orange) distributions log_10_ of the value of parameter are shown. Fixed *ad hoc* values were applied to the measurement model parameters (see Supplemental Table C (Case 2))

**Fig Q (part 2): Model parameters calibrated to Ordinal Dataset using Fixed *ad hoc* Measurement Model Parameters.** Parameters for aEARM were calibrated to ordinal values of tBID and cPARP abundance at every 60s interval. Prior (blue) and posterior (orange) distributions log_10_ of the value of parameter are shown. Fixed *ad hoc* values were applied to the measurement model parameters (see Supplemental Table C (Case 2))


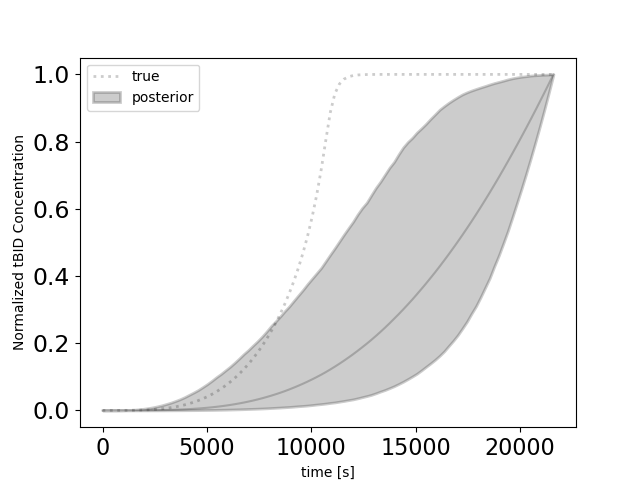


**Fig R: Predicted Bid truncation dynamics of aEARM trained to published fractional cell death data.** The 95% credible region of posterior predictions (shaded region) of normalized tBID dynamics for aEARM calibrated to published fractional cell death measurements by Wajant et al. (see *Methods Section 4*) [29]. The “true” line denotes dynamics of aEARM when its rate parameters are set to the maximum *a posteriori* parameter vector from the calibration by Ortega et al. This is the maximum *a posteriori* parameter vector that served as a “ground truth” parameters when generating the synthetic nonquantitative datasets (described in Methods Section 3), but it is not associated with published fractional cell death measurements by Wajant et al [29].

**
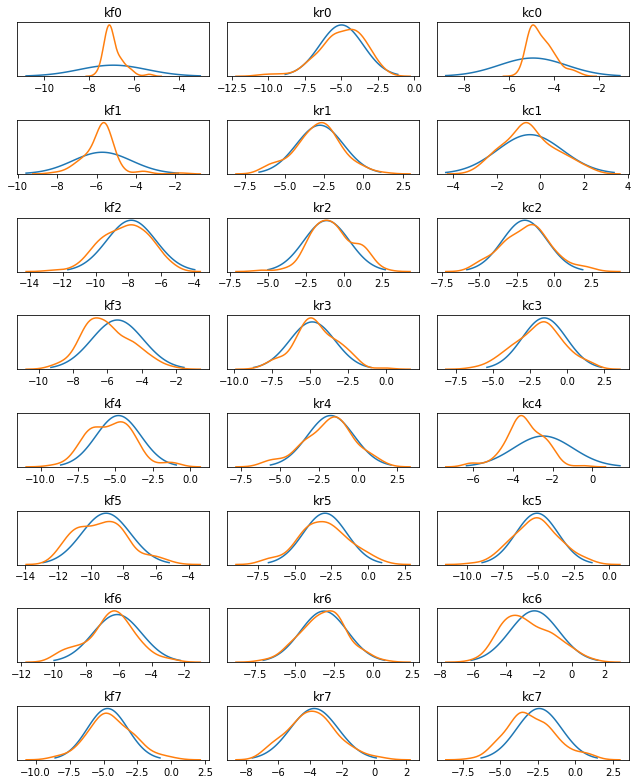
**

**Fig S (Also, see Fig 5): Model parameters calibrated to published fractional cell death data.** Parameters for aEARM were calibrated to published fractional cell death measurements by Wajant et al. (see *Methods Section 4*) [29]. Prior (blue) and posterior (orange) distributions of log_10_ of the value of parameter are shown

**
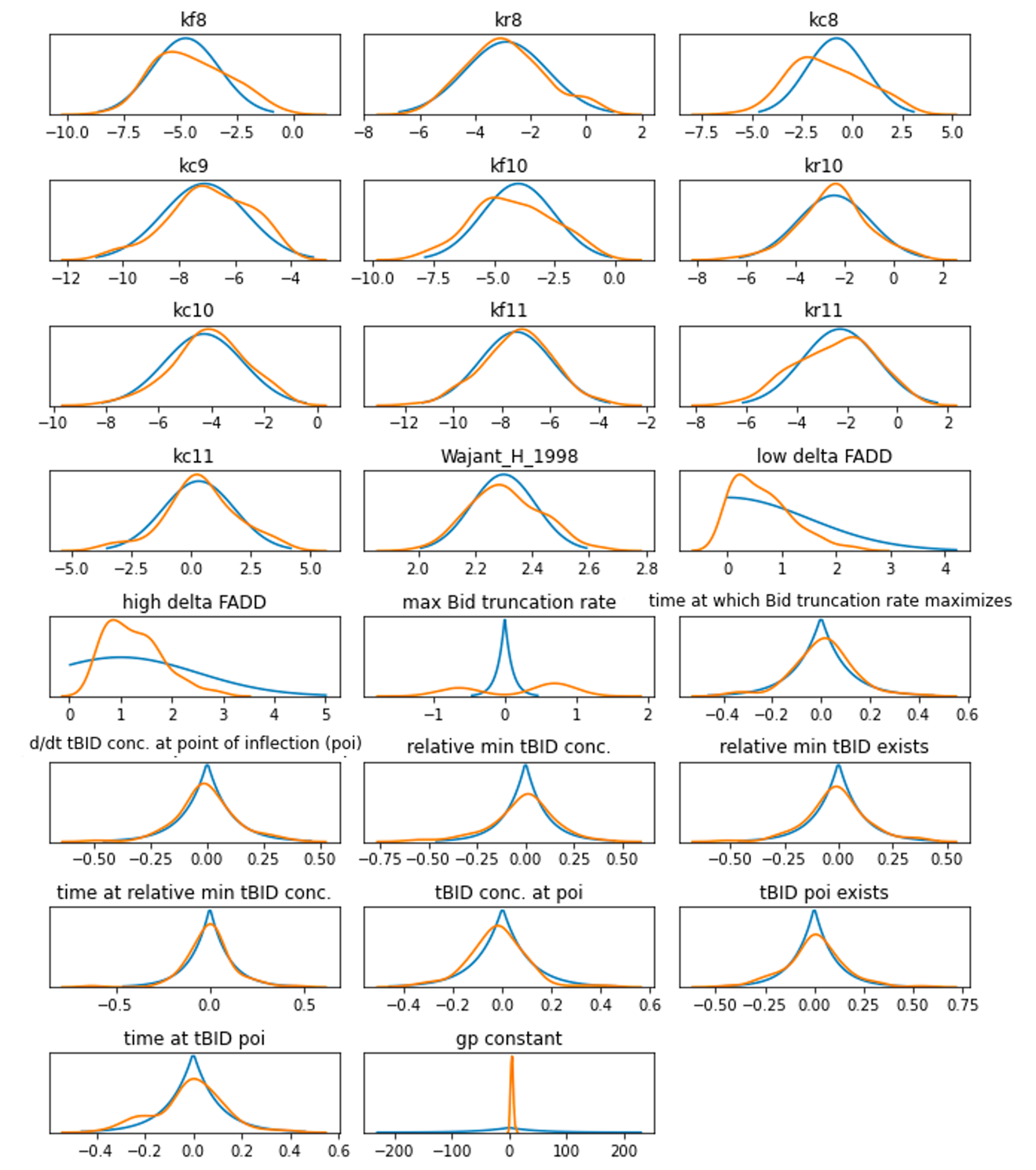
**

**Fig S (part 2) (Also, see Fig 5): Model parameters calibrated to published fractional cell death data.** Parameters for aEARM were calibrated to published fractional cell death measurements by Wajant et al. (see *Methods Section 4*) [29]^29^. Prior (blue) and posterior (orange) distributions log_10_ of the value of the aEARM parameter are shown. Prior and posterior distributions of the value of measurement model coefficients are also shown (these are not log-scale). We encoded initial TRAIL receptor concentrations ($R\_0$) as a free parameter “Wajant_H_1998” in EARM, to reflect potential differences in TRAIL sensitivity of the HeLa cells used in the experiments by Wajant et al, relative to the HeLa cells in experiments that produced the ICRP and ECRP fluorescence data. Since we did not calibrate aEARM to data from both sources, the prior and posterior for this parameter should not differ. The “gp_constant” is $\sigma_{f}^{2}$ from Eq. 20 (See methods section 2.4).


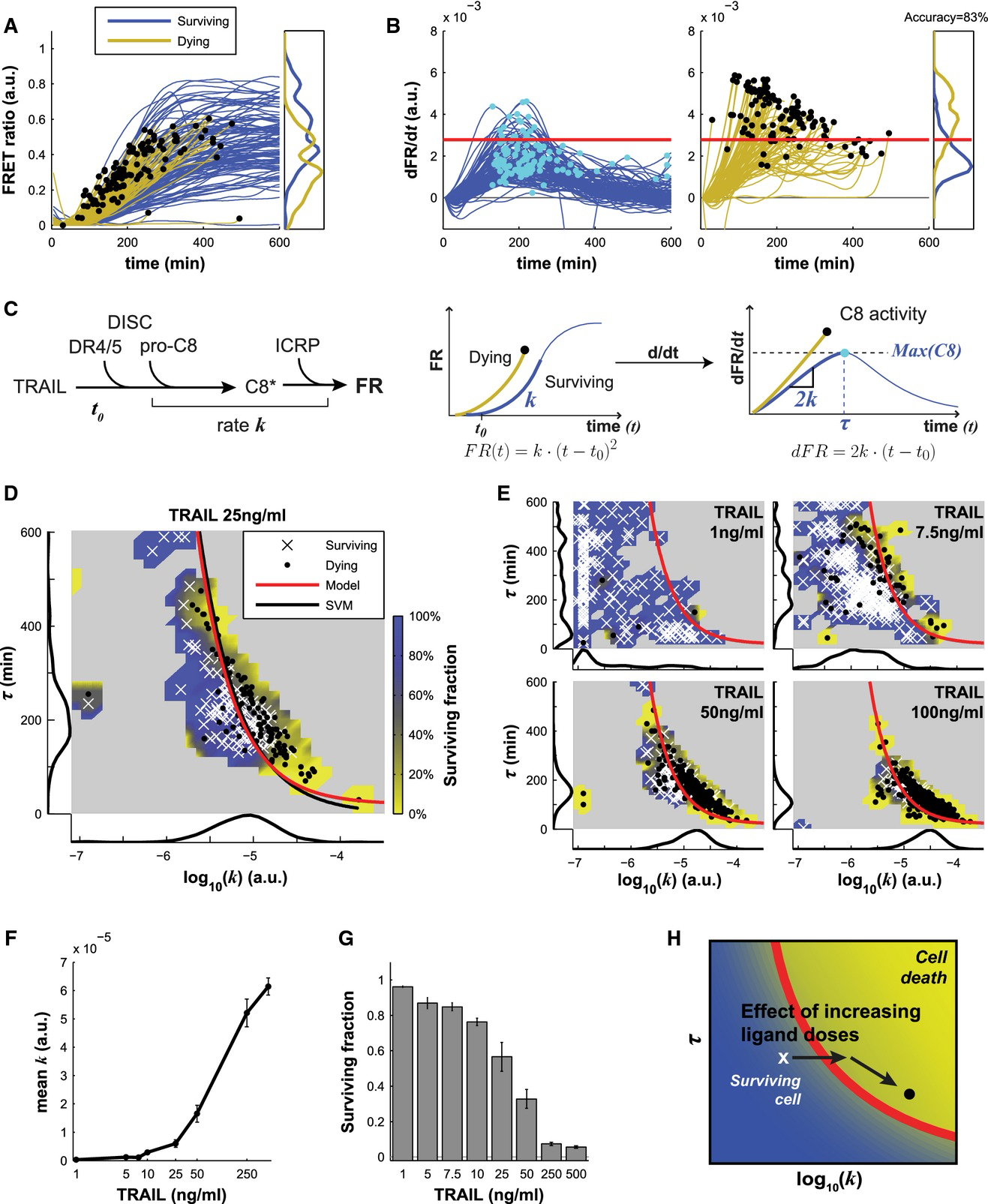


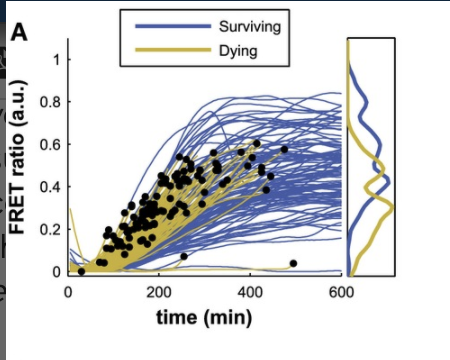


**
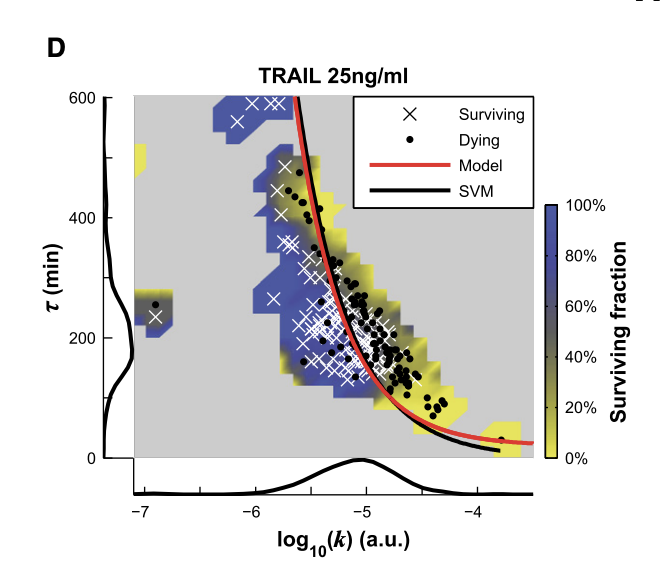
**

**Fig T: Reprinted from Roux et al. [1] caspase activity as a predictor of apoptosis in HeLa cells.**

A. The dynamics of Caspase activity, and by proxy rate of change in cleaved caspase substrate (e.g., Bid) concentrations, as indicated by a fluorescent indicator, served as features for predicting apoptosis (yellow) and survival (blue). B. The range of FRET ratio trajectories of HeLa cells treated with 25ng/mL TRAIL. C. The features dynamics of Caspase activity: $\log_{10} k$ which is a measure of maximum caspase activity (x-axis) and the time at which caspase activity maximizes $\tau$(y-axis) served as predictors of apoptosis and survival, as shown in the plot of surviving and apoptotic cells. The reprinted materials are from an open access article, in Molecular Systems Biology, under the terms of the Creative Commons Attribution 4.0 License, which permits use, distribution and reproduction in any medium, provided the original work is properly cited.

**References:**

1. Roux J, Hafner M, Bandara S, Sims JJ, Hudson H, Chai D, Sorger PK. Fractional killing arises from cell‐to‐cell variability in overcoming a caspase activity threshold. Molecular systems biology. 2015 May;11(5):803.
