# Supplementary material for: Model certainty in cellular network-driven processes with missing data
Source: PLoS Comput Biol. 2023 Apr 26;19(4):e1011004. doi: 10.1371/journal.pcbi.1011004 (PMC10166548; doi:10.1371/journal.pcbi.1011004)
Supplement: Attachment — Submitted filename: review_plos_cb.pdf [file pcbi.1011004.s003.pdf]

Review of M. W. Irvin, A. Ramanathan and C. F. Lopez, "Predictive uncertainty in mechanistic models of cellular processes calibrated to experimental data"

The authors develop a method that makes non-and semi-quantitative data accessible to calibration of mechanistic ODE models of biochemical networks. The central concept that the authors employ is that of a measurement model, which maps the variables of the mechanistic model to coarse-grained, phenomenological observables that can also represent non-and semi-quantitative data types. They construct generalized likelihoods that incorporate the measurement model and use those likelihoods for Bayesian model calibration.

I find the premise of the manuscript intriguing and consider the suggested method as potentially relevant.

However, I find that the overall presentation is lacking and the manuscript feels to me like a very rough draft that has been hastily put together. In my view, it is (too) sloppily written; the sheer number of issues–even though many of them are more minor–substantially hampers the readability and makes it unacceptably hard for a reader to assess and/or employ the authors' suggested methods. Another major concern is that the authors apply their method exclusively to a scaled-down version of the previously published EARM model. Therefore, it is difficult to judge whether the authors' method indeed provides a practically viable alternative to previously published approaches that the authors cite in the **Introduction**. Note that, for example, Ref. [13] considers a quite detailed model of yeast cell cycle control. Is the authors' method sufficiently efficient and hence capable of investigating a similarly detailed model? This is hard to say given the results presented in the manuscript. Therefore, it would greatly add to the manuscript if such a detailed analysis could be performed, perhaps even of the detailed model studied in Ref. [13], thus providing ample opportunity to compare the authors' approach with an alternative.

<u>Detailed comments:</u>

- In Box 1, **Calibration**, the authors do not mention priors or posteriors at all, but they state *Bayesian methods maximize likelihood functions* $\log \mathcal{L}(\hat{\mathbf{y}}|\boldsymbol{\theta})$... I find this statement slightly misleading. Would that not imply that only uniform priors are used?

- It is my understanding that the "ground truth" values of the rate constant parameters assembled in Supplementary (S.) Table 5 are the outcome of a Bayesian aEARM-model calibration to the fluorescence time-course data. In fact, the authors state in **Uncertainty associated with different data types in model calibration** (p. 5, line 209) *We then extracted a parameter vector from the fluorescence optimized data and used it as a baseline (reference) to generate ordinal datasets for tBID and other aEARM variables...* I would suggest to be more specific here: Do the authors use the MAP estimate to obtain the ground truth rate parameter values? Or do they use the Bayes estimate, that is, the posterior mean or

something else? Also, please provide the units that are used for the rate constants in S. Table 5. Or are non-dimensional units used? This is not clear to me; in Figs. 3A.) and 5, dimensional rate constant units are given.

- S. Figs. 4A and 4B display the rate constants' priors/posteriors of the calibration to the fluorescence data. I am somewhat confused about the priors. In **Model Calibration via Bayesian Inference** (p. 15), the choice of priors is briefly discussed and the authors state (line 682) *Rate parameters in aEARM were given independent log-normal prior probability functions with a location equal to the ground-truth parameter vector....* As far as I understand, the ground truth rate parameter values are the outcome of the Bayesian calibration. So how could they be used to construct the priors?

- Regarding the calibration to the ordinal datasets, I have the following questions/comments. According to its caption, S. Fig. 2 shows the priors and posteriors of the measurement model parameters. The corresponding legends seem to refer to the measurement model parameters as

  ```
  coefficients__tBID_blot__coef_,
  coefficients__tBID_blot__theta_1
  ```

  and so forth, but in other parts of the manuscript, such as **Measurement Models and Likelihood Functions**, and in the Supplementary Methods (S. Table 3) these parameters are referred to as α, β_j or α_{tBID}, $\boldsymbol{\beta}_{tBID}$ etc. Because the ground truth values given in S. Table 3 do not seem to be compatible with the values indicated by the dashed lines in the prior/posterior plots of S. Fig. 2, I assume that these plots actually show the distance terms θ_j that are briefly introduced in **Model Calibration via Bayesian Inference** (p. 16) rather than the actual measurement model parameters β_j. However, this is never clearly stated. So, if this is correct, please point this relation out more clearly and prominently. This also applies to Fig. 4 that, according to the caption and legends, shows plots of priors/posteriors of measurement model parameters as well, but again these plots seem to describe the distance terms. I find this confusing. Also, the use of θ_j to denote the distance terms is perhaps not the best choice, because it is already used to refer to the parameters of the mechanistic model, see for instance Eq. (12). In addition, it is not entirely clear to me to which part of the main manuscript S. Fig. 2 corresponds to. Because the priors look uniform, perhaps they relate to Fig. 4C.)? However, the posteriors look different, so it is hard to say. In general, I would suggest to make it easier to understand to which parts of the main manuscript the various Tables and Figures in the Supplementary Information relate to. I would also encourage to add an outline of the Supplementary Information's content and to add page numbers.

- A central concept that the authors use is the measurement model introduced in Box 1, Eq. (6). A function that is denoted in the same way,

namely f_M, appears in in Fig. 1, Eq. (3), suggesting it also represents a measurement model. However, in Eq. (6), f_M depends on $\mathbf{x}(t)$, the variables of the mechanistic model and maps to $y(t_i, \boldsymbol{\theta})$, while in Eq. (3), f_M is a function of y and $\mathbf{c}$ and maps to y_{obs}. What does $\mathbf{c}$ refer to? As far as I can see, this is never explained. Is it related to $\boldsymbol{\theta}$? Does y in Eq. (3) refer to the mechanistic model's variables $\mathbf{x}(t)$? How are $y_{obs}, y, y(t_i, \boldsymbol{\theta})$ related to each other? I find opportunities were here missed to explain this important concept more carefully and to keep at least the notation consistent. Also, the relation to the "probabilistic version" of the measurement model, Box 2, Eq. (9) should be explained in more detail.

- Fig. 1 seems to suggest that semi-quantitative data types play a special role, because only in this case a measurement model (f_M) is indicated. I would suggest to emphasize already in Fig. 1 that a measurement model can account for all four data types in a unified manner–even in the quantitative case it appears as the (trivial) identity map.

- In Box 1, **Mechanistic Models**, I find the given representation of an ODE (Eq. (5)) confusing; if there is no derivative, how can it be an ODE?

- Fig. 6: In Fig. 6E.) and the corresponding S. Fig. 20B somewhat cryptic names for parameters/features, such as

  `tBID_obs_p_poi_not_nan_0, gp_constant, Wajant_H_1998`

  appear. As far as I can see, those names are never introduced/explained.

- The main text refers to Fig. 2D, but there is no figure with this label: *...however, did not significantly constrain the posterior predictive regions of these dynamics (Figure 2D).* (p. 5, line 220). This is a recurrent issue, see below.

- S. Fig. 2: The caption refers to C., but only A., B. exist in the figure.

- S. Fig. 3: The caption refers to A., B., C., D. etc., but none of these labels appear in the actual figure. Some of the axes legends are cut-off and not readable. A reference is missing: *...and IC-RP fluorescence time-course (Fig 2A) data from [REF]).*

- There are two different figures in the Supplementary Methods referred to as S. Fig. 7A.

- S. Fig. 9: The caption again refers to A., B., C. D. that do not appear in the actual figure. Some of the axes legends are cut-off again.

- S. Fig. 10B: The dashed line in the prior/posterior plots of the measurement model parameters does not seem to be compatible with the ground truth values provided in S. Table 4. It looks as if in the corresponding plots the ground truth values flipped their sign. Again, the inconsistent

notation between S. Table 4 parameters and S. Fig. 10B legends does not help. Also, please introduce the population covariance term.

- S. Fig. 11B: The legends of the prior/posterior plots of the measurement model parameters are printed over each other and not readable. There is a similar issue in S. Fig. 12B.

- Virtually always the **Results** section refers to the **Methods** section only, without specifying the relevant subsection, such as **Integrating aEARM Dynamics**. This makes text navigation unecessarily hard.

- In my view, the **Methods** section in general, and, in particular, the **Measurement Models and Likelihood Functions** subsection, are too densely written. The manuscript's central concept, the measurement model and its incorporation into likelihoods should be introduced/explained in much more detail. For instance, the authors just mention the sequential model (p. 13, line 559). However, Ref. [40] that they cite discusses the sequential, cumulative and adjacent-category model as possible distinct classes of ordinal models. Why did the authors choose the sequential one over the two other alternatives for their application? Please motivate that choice.

- Eq. (14) refers to two distinct equations (see p. 14, p. 15). The same holds true for Eq. (15) (see p. 14, p. 15).

- Please explain the Gaussian process a little bit more detailed. What does $m(\mathbf{x})$ refer to? What is $\mathcal{D}$ on the lhs of Eq. (16)? What does K refer to on the rhs of Eq. (16)? Note that K is otherwise in **Measurement Models and Likelihood Functions** and **Generating Synthetic Datasets** (p. 13 and 14, respectively) used to denote the number of ordinal categories. But this does not seem to be the meaning of K in Eq. (16).

- Please explain the numbers $1.76, 6.02$ in Eq. (14) (the one on p. 15).

- The title of the manuscript changed from *Predictive uncertainty in mechanistic models of cellular processes calibrated to experimental data* (manuscript version without page numbers) to *Model certainty in cellular network-driven processes with missing data* (version with page numbers).
