# Supplementary material for: Model certainty in cellular network-driven processes with missing data
Source: PLoS Comput Biol. 2023 Apr 26;19(4):e1011004. doi: 10.1371/journal.pcbi.1011004 (PMC10166548; doi:10.1371/journal.pcbi.1011004)
Supplement: Attachment — Submitted filename: review_revision1.pdf [file pcbi.1011004.s005.pdf]

Review of M. W. Irvin, A. Ramanathan and C. F. Lopez, "Model certainty in cellular network-driven processes with missing data"

In my view, the authors addressed many of the raised issues in a satisfactory manner. However, I find the responses to Comment 2 and 17 somewhat lacking.
Comment 2:
While the overall presentation has been improved, in some parts the manuscript still appears to have been sloppily put together. In that regard it does not help that the revised manuscript (RM) and the revised manuscript with highlighted changes (RMH) do not seem to be consistent with each other. Please note that the RM lists the legends of Figure 1–Figure 7 (p.20–p.22). The actual figures that follow immediately after p.25 are also labeled as Figure 1–Figure 7. By contrast, the RMH lists the legends of Figure 1–Figure 5, and Box 1–Box 2 (p.20–p.24). As far as I can see, Box 1 and Box 2 are never mentioned in the RM. Presumably as a consequence, many of the figure references in the RM and the supplemental (S.) material do not seem to be correct. Further, the RM has an Author Summary while the RHM does not. All these issues should be carefully and thoroughly resolved.

There are a number of more minor issues that seem to appear in both the RM and the RMH. In the following, page and line numbers refer to the RM.

- Eq. 1: Please indicate that the rhs of an ODE typically depends on $\mathbf{x}(t)$ as well:
$$\dot{\mathbf{x}}(t) = f(\mathbf{x}, t, \theta),$$
rather than $\dot{\mathbf{x}}(t) = f(t, \theta)$.

- line 268: *We subsequently calibrated the parameters to fluorescence data;* Please provide a reference for the used data.

- line 303: *These data differ from synthetic ordinal and nominal datasets in that they lack a known reference to "ground truth" dynamics...* I am somewhat confused by this statement because S. Fig. 18 shows a time-course that is referred to as "true". Also, please provide the reference for the fractional cell death data in the main text.

- line 358: *Shown in Figure 5C-E, we represent this as a flexible assumption by encoding it in our priors (ii – iv) i.e., Cauchy distributions...* However, 5C's prior is a uniform rather than a Cauchy distribution.

- line 437: refers to Eq. 14. Should it be Eq. 19?

- line 445: refers to Eq. 16. Should it be Eq. 21?

- line 785: There seems to be a typo in Eq. 23. It should be
$$EBR = (SNR - 1.76)/6.02$$
.

- On occasion, y_{obs} is still used instead of $\hat{y}$ to refer to observed data. See line 897: *...can be compared to data $y_{obs}(t_i)$* and, by contrast, Eq. 6 that uses $\hat{y}$. Similarly, line 634: *...where data the, y_{obs},...* and, by contrast, Eq. 13 that uses $\hat{y}$.
- S. Table 3: *The prior and posterior plots below are of Θ_j.* There are no plots immediately below S. Table 3. Hence, please refer to the specific figures that contain the plots.
- S. Table 5: typo: *All kf use cell/copies-s as their units.*
- S. Fig. 7: legend of x-axis is partially cut off
- S. Figs. 18, 19A, 19B refer to Methods only. Please provide the specific section as well.
- The $\log(k) - \tau$ plot in S. Fig. 20 indicates a TRAIL dose of 50ng/ml. However, the figure caption and the main text on p. 10 only mention a 25ng/ml TRAIL dose.

The following sentences do not seem to be entirely correct:

- line 267: *We centered our the prior for...*
- line 273: *...and shown Figures 3B.*
- line 328: *...follow a well-establish and simple...*
- line 380: *...shows the predicted probabilities of the ordinal categories (as a function predicted cellular tBID content);*
- line 418: *To explore a practical application of the data-driven measurement model paradigm. We...*
- line 427: *...are indirect semi-quantitative measurements since lack a clear mapping between...*
- line 490: *...were sensitive to we encode assumptions in the measurement model,...*
- line 830: *To in encode empirical ordering constraints on the...*
- line 982: *...Guassian process...*
- line 984: *...Guassian process...*

<u>Comment 17:</u> In response to this comment the authors added on p. 14, line 659, *To represent the irreversible progression toward an apoptotic cell fate (and monotonic accumulation of apoptosis cell fate markers, tBID and cPARP), each ordinal constraint function is combined, using the sequential model...* And, on the same page, line 662, *The cumulative, and adjacent-category models may also be appropriate in other contexts.* I find this response somewhat too thin.

Especially the second sentence on the cumulative and adjacent-category models does not add much in my view and, as it stands, should be removed. Also, is the irreversible progression the reason why the authors chose the sequential model over the others? Or is it the monotonic accumulation of the markers? Please clarify and motivate/explain briefly the choice of the sequential model. Could the other two models used here? Why or why not (briefly)?
