# Supplementary material for: Model certainty in cellular network-driven processes with missing data
Source: PLoS Comput Biol. 2023 Apr 26;19(4):e1011004. doi: 10.1371/journal.pcbi.1011004 (PMC10166548; doi:10.1371/journal.pcbi.1011004)
Supplement: Attachment — Submitted filename: Reviewer_response_letter.docx [file pcbi.1011004.s004.docx]

Dear Editor,

Enclosed please find a revised version of our manuscript, entitled “*Model certainty in cellular network-driven processes with missing data.*”, by Michael W. Irvin, Arvind Ramanathan, and Carlos F. Lopez, to be considered for publication in *PLoS*. We thank the reviewers for carefully assessing our manuscript and providing constructive criticisms that have greatly improved the quality of our paper. In what follows, we present the reviewer comments (italics) followed by detailed replies to each suggestion along with changes to the manuscript as indicated by headings. The revised manuscript the amended material contain major revisions throughout. We have revised the Supplementary Materials document to further address some of the concerns raised by reviewers. We hope this version of the manuscript addresses reviewers’ comments, questions, and concerns, and look forward to your response.

Sincerely,

Carlos F. Lopez, PhD

(on behalf of all coauthors)

**Reviewer #1**

**Comment 1:**
*The authors make two significant contributions in my estimation. First, they extend the Bayesian inference methodological concepts of Ref. 12, wherein categorical observations of continuous variables are considered for use in model parameterization. In that work, the categorical observations are viewed as arising from a classifier function which has known threshold parameters. In this manuscript, the authors generalize, considering other types of functional relationships (GP/logistic) between observations and model/process variables and considering a wider range of combinations of observational data types. The authors argue that the function relating observations and model/process variables can be anything, such as a universal function approximator, and that the function may have different kinds of parameters besides thresholds, which can be learned jointly with model parameters. I think these are important ideas. The second contribution is that the authors provide another demonstration of how non-quantitative observations can be used to estimate model parameter values. There is some earlier work in this area but not much and the new demonstration differs from those provided earlier in several ways. The weakness of the demonstration is that it relies on synthetic data, but this is offset by using an empirically calibrated model to generate the synthetic datasets.*

**Author Response:**
We thank the reviewer for their positive comments and appreciate their appreciation for the innovations we sought to highlight in this work.

**Changes to Manuscript:**

None.

**Comment 2:**
*In the second paragraph of the Introduction, when discussing earlier work, it might be an improvement to include a clearer objective summary of earlier approaches and only then to present an evaluation of their limitations. It seems that the authors are criticizing the methodology of Ref. 12 (closest to the authors’ own work) because in that work thresholds are taken to be known. This is an assumption that can easily be relaxed in principle, so it is not a severe limitation. It is certainly fair to say that taking a threshold to be known can introduce bias, but it should probably be mentioned that taking some parameters to be fixed is commonly done in inference problems to make the inference problem practically solvable. It is stated that other earlier work also has limitations but these limitations are somewhat unclear to me from just reading the text. The limitations of all earlier methods discussed should be clarified, perhaps through the discussion of concrete examples.*

**Author Response:**

Thank you for highlighting this point as it was not our intention to appear critical! We agree that the use of fixed assumptions (e.g., like those used in Ref 12.) is a necessary part of the modeling process. The assumptions (or conversely free parameters) required by a measurement model reflects a bias-variance tradeoff and the inherent limitation of nonquantitative data. In the Result section, *Data-driven measurement model as an indicator of model bias*, we use Bayesian methods to calibrate a model to nonquantitative data using different fixed and free parameterizations of the measurement model. This provided an analysis of the impact of these assumptions on the accuracy and precision of the resulting models’ *a posteriori* prediction. Earlier work precludes easy application of Bayesian methods to mechanistic models incorperating nonquantitative data and therefore could not carryout this analysis. As the reviewer notes, simplifying assumptions are often unavoidable. We find that this consideration strengthens the value of our analysis.

**Changes to Manuscript:**

We edited the second paragraph extensively to reflect the impact of prior work more accurately and more clearly highlight the need for our contribution – particularly with the goal of removing any implied criticism. We reframed the contributions of Ref 12 and prior contributions to highlight the need for an understanding of how the choice of measurement model impacts the application of nonquantitative data, and the predictive power of the resulting calibrated mechanistic model. These same concepts are subsequently echoed throughout the text.

**Comment 3:**
*Consider defining the following terms in the text upon first use: nominal (binary categories?), ordinal (multiple categories?), semi-quantitative (e.g., relative measurements?), and quantitative. The discussion in the first paragraph of the Results section about the merits of the different data types could be made more interesting by saying more about the data. The discussion currently focuses on the value of the data, without saying much about the data.*

**Author Response:**

Thank you for suggesting this improvement. We find this feedback has been helpful to improve the quality of the manuscript.

**Changes to Manuscript:**

We significantly reworked the figures to address this important point. To attain this goal, we combined Figure 1, which listed examples of non-quantitative measurements, with Box 2, which discussed how such data would be used to train a mechanistic model, into a single “Box 2”. The edited version of Box 2 describes the measurement process and suggests different considerations to make when defining a measurement as nominal, ordinal or semi-quantitative. We also added content to the second paragraph of the Results section ***Contributions of different data types to mechanistic models of Apoptosis***, which expands further detail on these concepts. We hope this addresses the reviewers concern/suggestion.

**Comment 4:**
*The concepts of “measurand” and “measurement” should be more clearly defined when these terms are first brought up.*

**Author Response:**

We thank the reviewer for this comment. We find this suggestion improves the presentation of our work as it removes potential hurdles this concept could indeed pose to non-expert readers .

**Changes to Manuscript:**

We added the second paragraph of the Results section *Contributions of different data types to mechanistic models of Apoptosis*, which introduces and defines the measurand and measurement concepts, then refers the reader to Box 2 for further detail.

**Comment 5:**
*The comment “we introduce a concept from statistics, and social sciences: the measurement model” is a big overstatement. Measurement models, such as Equation (6) in Box 1, are commonly used by biological modelers. The overstatement is repeated in the Discussion: “Our work introduces the concept of measurement models to the mechanistic modeling paradigm.” I think what the authors are doing is using the well-known measurement model concept to connect non-quantitative observations to model/process variables, which is innovative. The authors should rephrase for precision and to avoid overstatement.*

**Author Response:**

We thank the reviewer for catching this! When the reviewer places it in this contex we understand how the statement that we “introduce measurement models from social sciences to mechanistic modeling paradigm” sounds like an overstatement. To address this point, we edited our wording to instead explain that biological modelers often use *implicit* measurement models (such as Equation 2) to train mechanistic models to fluorescence data, etc. We sought to both train our mechanistic models to nonquantitative data and *learn* interpretable relationships between the mechanistic model and supporting nonquantitative observations. It is this *learning objective* of the measurement model, which is more routine in social sciences, that inspires our approach in this work. We also now note, that our approach does not go as far as more complex measurement models (e.g., Structural Equation Models) as they do in other fields such as social sciences. Specifically while our approach enables the algorithm to learn a functional relationship between mechanistic model variables and phenotype (i.e., markers of the phenotype), we do not learn a causal relationship (i.e., drivers of the phenotype).

**Changes to Manuscript:**

We rephrase our mention of the measurement model. We use *implicit* measurement model for the traditional objective functions used for quantitative data, and *explicit* measurement model to denote our (and prior) approaches for integrating nonquantitative data.

**Comment 6:**

*I would like to see more information about the MCMC sampling, such as representative parameter and likelihood trace plots and pairs plots.*

**Author Response:**

We thank the reviewer for this request. We calibrated aEARM’s 30 parameters, plus additional measurement model parameters, to fourteen different datasets and/or measurement models. Each calibration generates 4 sets of parameter traces each.) these are available at the DOIs listed below. (It would be infeasible to plot 50 pages (35 GB) of traces). Note, the convergence of the PyDREAM method depends on the mixing of multiple MCMC traces and not any individual trace. We provide a table of Gelman-Rubin convergence statistics in the supplemental. The data is available in zenodo as described I the supplement and provided here for ease of access.

<https://doi.org/10.5281/zenodo.4768814>

<https://doi.org/10.5281/zenodo.4768842>

<https://doi.org/10.5281/zenodo.4768806>
<https://doi.org/10.5281/zenodo.4768370>

<https://doi.org/10.5281/zenodo.7007655>

**Changes to Manuscript:**

We more clearly summarize our approach in the results section and refer the reader to the Methods and Supplement sections for further details.

**Comment 7:**

*I am concerned about a sentence in the Abstract: “We find two orders of magnitude more ordinal (e.g. immunoblot) data are necessary to achieve accuracy comparable to quantitative (e.g. fluorescence) data.” It should probably be mentioned that this finding is likely to be problem-specific. The authors really only consider one demonstration problem. Consideration of many problems would be needed to start making general conclusions.*

**Author Response:**

Thank you for pointing out this accidental overstatement. We have changed the abstract to clarify this.

**Changes to Manuscript:**

We edited this sentence to qualify that these findings are specific to our apoptosis execution model.

**Reviewer #2:**

**Comment 1:**  *This paper adds to the growing but small community of modelers who are focused on the role of qualitative data as well as more model directed experimentation, something one might term model driven rather than a data driven approach. A model driven approach is I feel exactly the right approach. The current paper adds to the literature by conducting a more thorough analysis of the role of qualitative data. I was very interested to see a discussion of the concept of measurement model. The theory of development models and the role of data is not discussed very much, if at all in systems biology, and if anything, this paper will introduce the community to this more nuanced and important concept.*

*Overall, this was an interesting paper, its not a definitive analysis (as the authors admit) but it would be a useful addition to the literature and will hopefully stimulate further similar studies.*

**Author Response:**

We thank the reviewer for their perspective and are grateful for their appreciation of our work. As the reviewer notes, further investigation about the utility of different datasets in the scope of model directed experimentation is sorely needed! Following this positive comment, we further emphasized these points in our edited manuscript while also highlighting other contributions where possible.

**Changes to Manuscript:**

Added a mention of that the data-driven measurement model can guide model-driven experimentation, to the Conclusions at Line 554 to further emphasize this point, among other revisions.

**Comment 2:**  *Line 105. I would add some brief definitions of nominal, ordinal etc. to the text. I understand that Figure 1 describes these terms, but the caption is lengthy. Maybe something along the lines:*

*“nominal (data is categorized), ordinal (data is categorized and ranked),”. For semi-quantitative and quantitative, I’m don’t quite understand the difference. I would attempt to clarify these two terms more, but I recommend being succinct. Is semi-quantitative unit less data, e.g ratios and quantitative data with units? I wasn’t sure from the text.*

**Author Response:**

We thank the reviewer for their insightful comment. We added a paragraph at the beginning of the Results Section to address this point. We note however that the distinction between quantitative and semi-quantitative data varies from one paper to another, but the terms essentially describe the level of certainty provided in quantitative vs semi-quantitative measurements.

In this paper, we define quantitative measurements as interval scale measurements that can be directly compared to their measurand. For example, Mass-Spectrometry proteomics experiments report values (protein copies/unit volume) that directly represent their measurand (protein abundance). In contrast, semi-quantitative measurements are interval-scale measurements that indirectly represent their measurand. That is, they require additional empirical mathematical transformations to connect the measurement and measurand. For example, Fluorescence experiments report fluorescence intensity values (arbitrary units, photons, detector voltage, etc.) that are related to protein abundance via some unknown or empirically determined function.

The reviewer’s advice inspired us to combine Figure 1 and Box 2 so that our definition and use of these measurement types can be more clearly conveyed to the readers.

**Changes to Manuscript:**

We combined Figure 1 (which listed examples of non-quantitative measurements) and Box 2 (which discussed how such data would be used to train a mechanistic model) into a single box: Box 2. The edited version of Box 2 describes the measurement process and suggests different considerations to make when defining a measurement as nominal, ordinal or semi-quantitative. We also added the second paragraph of the Results section *Contributions of different data types to mechanistic models of Apoptosis*, which provides further detail.

**Comment 3:**  *Line 144: “The model was calibrated to above fluorescence data”, missing word somewhere?*

**Author Response:**

Thank you for pointing this out.

**Changes to Manuscript:**
Edited Line 144 (Line 188 in the revised manuscript) to: “The model was calibrated to the above fluorescence data”.

**Comment 4:**  *Line 249: “As described in Methods, we added a synthetic 249 dataset containing 61 ordinal time-course measurements” I didn’t immediately quite understand how time-course measurements can be an ordinal data type (this is briefly explained in the methods section). Could the authors point to the method section (line 631 in the manuscript I assume?) where this is described, for example: As described in the Methods x.y, we added a synthetic…”. The authors indicate in the methods section that: “These time-courses were converted to ordinal time-course datasets”, from reading the text I assume the time course data was just turned into a set of ranked data but without a quantitative time dimension?*

**Author Response:**

We thank the reviewer for pointing out a potential source of confusion. The ordinal datasets had ordinal values of protein abundance (e.g. Level 1, Level 2, Level 3…) at discreet time points (30s, 60s, 90s…). They are ordinal (despite being time course data) because protein abundance is expressed as ordinal levels. That is, the values for protein abundance provide some insight about the ordinality of the data but are devoid of quantitative interpretation. As the reviewer notes, one could also further coarse grain the time coordinate but this is something that is typically reported in e.g. time-course immunoblot data.

**Changes to Manuscript:**
We added a sentence at Line 185 to explain how time-course measurements can also be ordinal and thus clarify the point raised. We also refer the reader (at Line 249 (Line 322 in the revised manuscript) to Methods Section 3 for further detail.

**Comment 5:**
*I recommend that wherever the main text says “See Methods” or similar, I would be explicit about where in the methods section the reader to go to, this would greatly help the reader.*

**Author Response:**

Thank you suggesting this improvement.

**Changes to Manuscript:**
Classified the Methods section into subsections and updated all references to the Methods to references to Methods X.Y.

**Comment 6:**  *Line 269-270: “but this time we replaced the free parameters in the measurement model fixed a priori parameterizations”, text doesn’t read correctly? Now sure what is being said here.*

**Author Response:**
We thank the reviewer for highlighting a potentially confusing sentence. In this example, the measurement model parameters are preset values and not calibrated to data.

**Changes to Manuscript:**

We edited Line 270 (Line 322 in the revised manuscript) to clarify that the parameters were preset and not calibrated to data.

**Comment 7:**
*Conclusion: I would include in the conclusion a series of bullets points outlining the basic recommendations and results obtained from the work. I think many readers might find it difficult to extract the key conclusions as the text is fairly dense. I think there are perhaps three primary conclusions from the work, these should be corrected or reworded if I am inaccurate here: 1) A lot more ordinal data is required to constrain a model; 2) Prior assumptions about parameter values can bias a model; 3) Use the model to devise the most profitable data to collect.*

**Author Response:**

We thank the reviewer for their thoughtful insights about our paper! The reviewer distils the main points of the paper accurately.

As pointed out by the reviewer, *“3) Use the model to devise the most profitable data to collect.”*, this is indeed correct, but this also depends on the measurement models being formulated such that they can *learn* interpretable relationships between the model/process variables and the observations. This learned information can then be applied beyond model fitting (e.g., designing experiments to devise more profitable data collection).

**Changes to Manuscript:**
We more clearly and concisely enumerate the main contributions of our work in the first paragraph of the conclusions section.

**Comment 8:**  *Line 766: Typo in “We Consider”, Figure 1*

**Author Response:**

Thank you.

**Changes to Manuscript:**
We corrected the typo.

**Comment 8:**
*Last but not least, the GitHub link for the source code appears to take one to a 402 page.*

**Author Response:**

None

**Changes to Manuscript:**
We have made the GitHub repositories public.

**Reviewer #3**

**Comment 1:**

*The authors develop a method that makes non-and semi-quantitative data accessible to calibration of mechanistic ODE models of biochemical networks. The central concept that the authors employ is that of a measurement model, which maps the variables of the mechanistic model to coarse-grained, phenomenological observables that can also represent non-and semi-quantitative data types. They construct generalized likelihoods that incorporate the measurement model and use those likelihoods for Bayesian model calibration.*

*I find the premise of the manuscript intriguing and consider the suggested method as potentially relevant.*

**Author Response:**

We thank the reviewer for the positive feedback.

**Changes to Manuscript:**

None

**Comment 2:**

*However, I find that the overall presentation is lacking and the manuscript feels to me like a very rough draft that has been hastily put together. In my view, it is (too) sloppily written; the sheer number of issues–even though many of them are more minor–substantially hampers the readability and makes it unacceptably hard for a reader to assess and/or employ the authors’ suggested methods.*

**Author Response:**

We thank the reviewer for their feedback. Following the reviewers’ comments, we have shared the paper with colleagues, obtained feedback, and worked to improve the readability.

**Changes to Manuscript:**

Stylistic and other changes to improve readability and consistency.

**Comment 3:**

*Another major concern is that the authors apply their method exclusively to a scaled-down version of the previously published EARM model. Therefore, it is difficult to judge whether the authors’ method indeed provides a practically viable alternative to previously published approaches that the authors cite in the Introduction. Note that, for example, Ref. [13] considers a quite detailed model of yeast cell cycle control. Is the authors’ method sufficiently efficient and hence capable of investigating a similarly detailed model? This is hard to say given the results presented in the manuscript. Therefore, it would greatly add to the manuscript if such a detailed analysis could be performed, perhaps even of the detailed model studied in Ref. [13], thus providing ample opportunity to compare the authors’ approach with an alternative.*

**Author Response:**

We thank the reviewer for their insightful feedback. The perceived weaknesses may be the result of a misunderstanding. We do not intend to compete with or replace methods in Ref [10-13]. Rather, our goal is to raise considerations about how assumptions in the data and measurement model can cause biases on calibrated mechanistic models. We deliberately chose a smaller model where all parameters could be identified by the limited available data. As shown by others in our community, larger models typically possess unidentifiable parameters that could confound our basic finding. We do agree however that further work is needed to directly compare the model calibration methods of [10-13], but this would be outside the scope of the manuscript.

**Changes to Manuscript:**

We significantly revised the second and third paragraph of the Introduction section to clearly state the goals of our work.

**Comment 4:**

*In Box 1, Calibration, the authors do not mention priors or posteriors at all, but they state Bayesian methods maximize likelihood functions* $\log L\left( \hat{y} \right|\theta)$*... I find this statement slightly misleading. Would that not imply that only uniform priors are used?*

**Author Response:**

We thank the reviewer for pointing this out. We examine the effect of different prior parameterizations on our measurement model in the Results Section *Data-driven measurement model as an indicator of model bias*. We further highlight the kinds of priors used in the Methods section and supplemental.

**Changes to Manuscript:**

We added a new Box 1, where we clearly define parameter priors and refer readers to the Methods section where these are further described.

**Comment 5:**

*It is my understanding that the “ground truth” values of the rate constant parameters assembled in Supplementary (S.) Table 5 are the outcome of a Bayesian aEARM-model calibration to the fluorescence time-course data. In fact, the authors state in Uncertainty associated with different data types in model calibration (p. 5, line 209) “We then extracted a parameter vector from the fluorescence optimized data and used it as a baseline (reference) to generate ordinal datasets for tBID and other aEARM variables”... I would suggest to be more specific here: Do the authors use the MAP estimate to obtain the ground truth rate parameter values? Or do they use the Bayes estimate, that is, the posterior mean or something else?*

*Also, please provide the units that are used for the rate constants in S. Table 5. Or are non-dimensional units used? This is not clear to me; in Figs. 3A.) and 5, dimensional rate constant units are given.*

**Author Response:**

We thank the reviewer for pointing this out this potential source of confusion. Understanding that this was not clear we significantly edited our explanation. The model was previously calibrated to fluorescence data in Ortega et al. [Ref 23]. We extract the MAP estimate from this calibration and use it as our “ground-truth” for the calibrations in this study.

The units for all kf values were $cell \times copies^{-1} \times second^{-1}$ .

The units for all kr and kc values were $second^{-1}$.

**Changes to Manuscript:**

We clarified what we mean by “ground-truth” parameters in the 3^rd^ paragraph of the *Results* and in *Methods Sections* *1 and 5*. We also made sure units were reported clearly in S. Table 5**.**

**Comment 4:**

*S. Figs. 4A and 4B display the rate constants’ priors/posteriors of the calibration to the fluorescence data. I am somewhat confused about the priors. In Model Calibration via Bayesian Inference (p. 15), the choice of priors is briefly discussed and the authors state (line 682) Rate parameters in aEARM were given independent log-normal prior probability functions with a location equal to the ground-truth parameter vector.... As far as I understand, the ground truth rate parameter values are the outcome of the Bayesian calibration. So how could they be used to construct the priors?*

**Author Response:**

We thank the reviewer for pointing this potential source for confusion. The aEARM first was calibrated using a log-normal prior with log-variance of 1.5 and log-mean of -6, -3, and 0 for the kf, kr and kc of each reaction respectively (each reaction except those associated with complexes). This calibration was originally presented in Ortega O. et al. as cited in our paper.

**Changes to Manuscript:**

We clarified mentions of the “ground-truth” parameters in the 3^rd^ paragraph of the *Results* and in *Methods Sections* *1 and 5* to convey this more clearly.

**Comment 5:**

*Regarding the calibration to the ordinal datasets, I have the following questions/comments. According to its caption, S. Fig. 2 shows the priors and posteriors of the measurement model parameters. The corresponding legends seem to refer to the measurement model parameters as coefficients__tBID_blot__coef_, coefficients__tBID_blot__theta_1 and so forth, but in other parts of the manuscript, such as Measurement Models and Likelihood Functions, and in the Supplementary Methods (S. Table 3) these parameters are referred to as α, β_j or α_tBID, β_tBID etc. Because the ground truth values given in S. Table 3 do not seem to be compatible with the values indicated by the dashed lines in the prior/posterior plots of S. Fig. 2, I assume that these plots actually show the distance terms θ_j that are briefly introduced in Model Calibration via Bayesian Inference (p. 16) rather than the actual measurement model parameters β_j . However, this is never clearly stated. So, if this is correct, please point this relation out more clearly and prominently.*

*This also applies to Fig. 4 that, according to the caption and legends, shows plots of priors/posteriors of measurement model parameters as well, but again these plots seem to describe the distance terms. I find this confusing. Also, the use of θ_j to denote the distance terms is perhaps not the best choice, because it is already used to refer to the parameters of the mechanistic model, see for instance Eq. (12).*

*In addition, it is not entirely clear to me to which part of the main manuscript S. Fig. 2 corresponds to. Because the priors look uniform, perhaps they relate to Fig. 4C.)? However, the posteriors look different, so it is hard to say. In general, I would suggest to make it easier to understand to which parts of the main manuscript the various Tables and Figures in the Supplementary Information relate to. I would also encourage to add an outline of the Supplementary Information’s content and to add page numbers*

**Author Response:**

We thank the reviewers for highlighting the lack of clarity in our explanation for parameterizations used in the measurement model (and associated plots). The variables “*coefficients__tBID_blot__theta_1”,” coefficients__tBID_blot__theta_2”* etc. in the supplemental figures show values for the distance terms $\theta_{1}, \theta_{2}$, etc. S. Fig 2 corresponds to the Box 2B in the original manuscript. Box 2B was a plot of the probability of each ordinal category for tBID predicted by the measurement model which was calibrated to ordinal data while hold the mechanistic model parameters fixed at a ground truth parameterization. We generated this figure to explain our measurement model. We think that the new schematic in our revised Box 1 captures these concepts more clearly and removed the problematic plot from Box 2 to avoid confusion.

**Changes to Manuscript:**

We edited *Method section 2.2,* which introduces our calibration to ordinal data, and clearly explain the relationship between $\theta_{j}$ and $\beta_{j}$. We also added ground truth values for $\theta_{j}$ to Supplemental Table 3, and text explaining that $\theta_{j, tBID}$ corresponds to “*coefficients__tBID_blot__theta_j”* and so on. We also added a note to the caption of Figure 4 further explaining this relation. We also removed S. Fig 2 as it no longer relates to plots in the main manuscript. We also added a table of contents section to the supplement.

**Comment 6:**

*A central concept that the authors use is the measurement model introduced in Box 1, Eq. (6). A function that is denoted in the same way, namely* $f_{M}$*, appears in in Fig. 1, Eq. (3), suggesting it also represents a measurement model. However, in Eq. (6),* $f_{M}$*, depends on x(t), the variables of the mechanistic model and maps to y(t_i , θ), while in Eq. (3),* $f_{M}$*, is a function of y and c and maps to y_obs. What does c refer to? As far as I can see, this is never explained. Is it related to θ? Does y in Eq. (3) refer to the mechanistic model’s variables x(t)? How are yobs, y, y(t_i , θ) related to each other?*

*I find opportunities were here missed to explain this important concept more carefully and to keep at least the notation consistent. Also, the relation to the” probabilistic version” of the measurement model, Box 2, Eq. (9) should be explained in more detail.*

**Author Response:**

We thank the reviewer for pointing this source of confusion. This was an accidental omission on our part. We have now edited the equations in the manuscript and figures for greater consistency.

By *probabilistic* we imply that the measurement model predicts a probability of a categorical value rather than predicting the categorical value itself. We describe this Box 1 Eq. 3 and in the Methods section.

**Changes to Manuscript:**

We edited the equations in the manuscript and figures for greater consistency. We combined Box 2 and Figure 1 into a single Box 2 and redid Box 1 to more clearly express the concept of the measurement model and how it different kinds of measurements entail different measurement models.

**Comment 7:**

*Fig. 1 seems to suggest that semi-quantitative data types play a special role, because only in this case a measurement model (*$f_{M}$*), is indicated. I would suggest to emphasize already in Fig. 1 that a measurement model can account for all four data types in a unified manner–even in the quantitative case it appears as the (trivial) identity map.*

**Author Response:**

The reviewer makes a great point. We are grateful that the reviewer appreciates that a measurement model can be defined for any data type as this is an important contribution in our paper. We edited Box 1 to emphasize this point more clearly.

**Changes to Manuscript:**

We edited Box 1 to show explicit definitions of a measurement model of the different kinds of measurements we examined in this paper.

**Comment 8:**

*In Box 1, Mechanistic Models, I find the given representation of an ODE (Eq. (5)) confusing; if there is no derivative, how can it be an ODE?*

**Author Response:**

This was an accidental statement on our part. We thank the reviewer for pointing this out! Our approach is agnostic to the mathematical representation. We have now edited Box 1 to clearly reflect this point.

**Changes to Manuscript:**

Change the representation of mechanistic models to Eq. 1, which explicitly defines an ODE.

**Comment 9:**

*Fig. 6: In Fig. 6E.) and the corresponding S. Fig. 20B somewhat cryptic names for parameters/features, such as tBID_obs_p_poi_not_nan_0, gp_constant, Wajant_H_1998 appear. As far as I can see, those names are never introduced/explained.*

**Author Response:**

We thank the reviewer for their attention to detail!

**Changes to Manuscript:**

We revised the Methods section to include a detailed description about the parameters listed in Fig 6E and S. Fig 20B. We also renamed the parameter/features so that they are consistent with the text in the manuscript.

**Comment 10:**

*The main text refers to Fig. 2D, but there is no figure with this label: ...however, did not significantly constrain the posterior predictive regions of these dynamics (Figure 2D). (p. 5, line 220). This is a recurrent issue, see below.*

**Author Response:**

We thank the reviewer for pointing out this accidental mistake.

**Changes to Manuscript:**

Edited Line 291 (in the revised manuscript) to reference the correct plot.

**Comment 11:**

*S. Fig. 2: The caption refers to C., but only A., B. exist in the figure.*

**Author Response:**

None

**Changes to Manuscript:**

Removed S. Fig 2 since it no longer relates to the revised manuscript.

**Comment 12:**

*S. Fig. 3: The caption refers to A., B., C., D. etc., but none of these labels appear in the actual figure. Some of the axes legends are cut-off and not readable. A reference is missing: ...and IC-RP fluorescence time-course (Fig 2A) data from [REF]).*

**Author Response:**

We thank the reviewer for finding this place where clarification was needed.

**Changes to Manuscript:**

We edited S. Fig 3 for clarity and added the reference as suggested.

**Comment 13:**

*There are two different figures in the Supplementary Methods referred to as S. Fig. 7A. • S. Fig. 9: The caption again refers to A., B., C. D. that do not appear in the actual figure. Some of the axes legends are cut-off again.*

**Author Response:**

Thank you for pointing this out. The caption in S. Fig. 9 was referencing subsections of a Figure in the main text.

**Changes to Manuscript:**

Renumbered the figures in Supplemental Materials. We rephrased the caption in S. Fig. 9 for clarity.

**Comment 14:**

*S. Fig. 10B: The dashed line in the prior/posterior plots of the measurement model parameters does not seem to be compatible with the ground truth values provided in S. Table 4. It looks as if in the corresponding plots the ground truth values flipped their sign. Again, the inconsistent notation between S. Table 4 parameters and S. Fig. 10B legends does not help. Also, please introduce the population covariance term*

**Author Response:**

Thank you for pointing this out. The ground-truth parameters (used to generate the cell death vs. survival dataset) had an unaccounted negative. We corrected the ground truth parameterization in S. Table 4

**Changes to Manuscript:**

We corrected the ground truth parameterization in S. Table 4.

**Comment 15:**

*S. Fig. 11B: The legends of the prior/posterior plots of the measurement model parameters are printed over each other and not readable. There is a similar issue in S. Fig. 12B.*

**Author Response:**

Thank you for pointing this out.

**Changes to Manuscript:**

Corrected the legends and captions for these figures.

**Comment 16:**

*Virtually always the Results section refers to the Methods section only, without specifying the relevant subsection, such as Integrating aEARM Dynamics. This makes text navigation unecessarily hard.*

**Author Response:**

We thank the reviewer for pointing this out.

**Changes to Manuscript:**
Divided the Methods section into subsections and updated all references to the Methods to references to Methods X.Y.

**Comment 17:**

*In my view, the Methods section in general, and, in particular, the Measurement Models and Likelihood Functions subsection, are too densely written. The manuscript’s central concept, the measurement model and its incorporation into likelihoods should be introduced/explained in much more detail. For instance, the authors just mention the sequential model (p. 13, line 559). However, Ref. [40] that they cite discusses the sequential, cumulative and adjacent-category model as possible distinct classes of ordinal models. Why did the authors choose the sequential one over the two other alternatives for their application? Please motivate that choice.*

**Author Response:**

We thank the reviewer for pointing this out. We agree the measurement model and its incorporation into likelihoods is a central concept of the manuscript and have edited the manuscript extensively to better convey this. We were a bit confused by the suggestion that it was “too dense” but that it also needed “much more detail”. We have done our best to make the flow of the explanation more clear in hopes that this improves readability as suggested by the reviewer.

Since we are modeling the dynamics of an irreversible progression toward an apoptotic cell fate (and the monotonic accumulation of apoptosis cell fate markers). We selected the sequential model.

**Changes to Manuscript:**

Significantly revised the methods section to provide more detailed explanation of our work.

**Comment 18:**

*Eq. (14) refers to two distinct equations (see p. 14, p. 15). The same holds true for Eq. (15) (see p. 14, p. 15).*

**Author Response:**

We thank the reviewer for pointing this out.

**Changes to Manuscript:**

We renumbered the Equations.

**Comment 19:**

*Please explain the Gaussian process a little bit more detailed. What does m(x) refer to? What is D on the lhs of Eq. (16)? What does K refer to on the rhs of Eq. (16)? Note that K is otherwise in Measurement Models and Likelihood Functions and Generating Synthetic Datasets (p. 13 and 14, respectively) used to denote the number of ordinal categories. But this does not seem to be the meaning of K in Eq. (16).*

**Author Response:**

We thank the reviewer for pointing this out.

**Changes to Manuscript:**

We significantly revised *Methods Section 2.4 Calibration to Cell Viability Data* to provide more detail about the Gaussian process model.

We changed $K$ in the equations referencing ordinal categories to $J$.

**Comment 20:**

*Please explain the numbers 1.76, 6.02 in Eq. (14) (the one on p. 15).*

**Author Response:**

These constants are standard to effective bit resolution (EBR) equations and explained in the reference.

**Changes to Manuscript:**

Add Line 844 in the manuscript explaining the equation.

**Comment 21:**

*The title of the manuscript changed from Predictive uncertainty in mechanistic models of cellular processes calibrated to experimental data (manuscript version without page numbers) to Model certainty in cellular network driven processes with missing data (version with page numbers).*

**Author Response:**

We thank the reviewer for pointing this out. We did update the title of the paper to reflect the contents of the paper more accurately.

**Changes to the Manuscript**

Kept the new title and included page numbers
