# Supplementary material for: Model certainty in cellular network-driven processes with missing data
Source: PLoS Comput Biol. 2023 Apr 26;19(4):e1011004. doi: 10.1371/journal.pcbi.1011004 (PMC10166548; doi:10.1371/journal.pcbi.1011004)
Supplement: Attachment — Submitted filename: Reviewer Response Letter.docx [file pcbi.1011004.s006.docx]

Dear Editor,

Enclosed please find a revised version of our manuscript, entitled “*Model certainty in cellular network-driven processes with missing data.*”, by Michael W. Irvin, Arvind Ramanathan, and Carlos F. Lopez, to be considered for publication in *PLoS*. We thank the reviewers for carefully assessing and providing constructive criticisms to our manuscript. In what follows, we present responses to the additional suggestions and concerns expressed by Reviewer 3. The reviewer comments (italics) are followed by detailed replies to each comment along with changes made to the manuscript as indicated by each heading. The updated manuscript and supplementary materials contain revisions as needed. We hope this version of the manuscript addresses the concerns raised by Reviewer 3 and look forward to your response.

Sincerely,

Carlos F. Lopez, PhD

(On behalf of all coauthors)

**Comment 1:**

*Comment 2: While the overall presentation has been improved, in some parts the manuscript still appears to have been sloppily put together. In that regard it does not help that the revised manuscript (RM) and the revised manuscript with highlighted changes (RMH) do not seem to be consistent with each other. Please note that the RM lists the legends of Figure 1–Figure 7 (p.20–p.22). The actual figures that follow immediately after p.25 are also labeled as Figure 1–Figure 7. By contrast, the RMH lists the legends of Figure 1–Figure 5, and Box 1–Box 2 (p.20–p.24). As far as I can see, Box 1 and Box 2 are never mentioned in the RM. Presumably as a consequence, many of the figure references in the RM and the supplemental (S.) material do not seem to be correct. Further, the RM has an Author Summary while the RHM does not. All these issues should be carefully and thoroughly resolved.*

*There are a number of more minor issues that seem to appear in both the RM and the RMH. In the following, page and line numbers refer to the RM.*

**Author Response:**

We thank the reviewer for their feedback and their recommended edits.

**Changes to Manuscript:**

Addressed the recommended changes (see next comments), and the discrepancy between the revised manuscript and the revised manuscript with highlighted changes.

**Comment 2:**

*Eq. 1: Please indicate that the rhs of an ODE typically depends on x(t)*

*as well:*

*x ̇(t) = f(x, t, θ), rather than x ̇(t) = f(t, θ).*

**Author Response:**

We thank the reviewer for pointing this out.

**Changes to Manuscript:**

Updated Eq. 1

**Comment 3:**

*Line 268: We subsequently calibrated the parameters to fluorescence data; Please provide a reference for the used data.*

**Author Response:**

We thank the reviewer for this recommended edit, as it improves the clarity of this section. We calibrate aEARM to the fluorescence dataset by Ortega et al. We extracted the maximum *a posteriori* (MAP) parameter vector, centered our prior for the aEARM rate parameters on this MAP parameter vector. We then, calibrated aEARM rate parameters to the *same* dataset in Ortega et al. This dataset was originally presented in Albeck, et al. 2008 (ref. 21).

**Changes to Manuscript:**

Edited the Line 268 to more clearly reference the fluorescence dataset used in our study.

**Comment 4:**

*Line 303: These data differ from synthetic ordinal and nominal datasets in that they lack a known reference to “ground truth” dynamics... I am somewhat confused by this statement because S. Fig. 18 shows a time-course that is referred to as “true”. Also, please provide the reference for the fractional cell death data in the main text.*

**Author Response:**

We thank the reviewer for pointing this out. The “true” dynamics in S. Fig. 18 are those described by the maximum *a posteriori* parameter vector what we extracted from the calibrations by Ortega et al. Unlike the synthetic ordinal and nominal datasets, that were generated from this maximum *a posteriori* “ground-truth” parameter vector, the fraction cell death data were generated via experiments by Wajant et al. and not associated with the “ground-truth”.

**Changes to Manuscript:**

Edited the caption to S. Fig. 18 to describe the plots more clearly.

**Comment 5:**

*Line 358: Shown in Figure 5C-E, we represent this as a flexible assumption by encoding it in our priors (ii – iv) i.e., Cauchy distributions... However, 5C’s prior is a uniform rather than a Cauchy distribution.*

**Author Response:**

We thank the reviewer for pointing this out.

**Changes to Manuscript:**

Edited Line 358 to describe these priors more accurately.

**Comment 6:**

*Line 437: refers to Eq. 14. Should it be Eq. 19?*

*Line 445: refers to Eq. 16. Should it be Eq. 21?*

**Author Response:**

We thank the reviewer for pointing this out.

**Changes to Manuscript:**

Corrected the referenced lines.

**Comment 7:**

*Line 785: There seems to be a typo in Eq. 23. It should be EBR = (SNR − 1.76)/6.02?*

**Author Response:**

We thank the reviewer for pointing this out. Eq. 24 makes this confusing since it presents the noise term, $q_{0.95}\text{rms}(d)$, in the numerator.

**Changes to Manuscript:**

Corrected Eq. 23 and Eq. 24.

**Comment 8:**

*On occasion, yobs is still used instead of ˆy to refer to observed data. See line 897: ...can be compared to data yobs(ti) and, by contrast, Eq. 6 that uses ˆy. Similarly, line 634: ...where data the, yobs,... and, by contrast, Eq. 13 that uses ˆy.*

**Author Response:**

We thank the reviewer for pointing this out.

**Changes to Manuscript:**

Corrected such discrepancies where they occurred.

**Comment 9:**

*S. Table 3: The prior and posterior plots below are of Θj . There are no plots immediately below S. Table 3. Hence, please refer to the specific figures that contain the plots.*

**Author Response:**

We thank the reviewer for this suggestion.

**Changes to Manuscript:**

Edited S. Table 3 with more specific references to figures.

**Comment 10:**

*S. Table 5: typo: All kf use cell/copies-s as their units.*

**Author Response:**

We thank the reviewer for this pointing this out.

**Changes to Manuscript:**

Corrected typo.

**Comment 11:**

*• S. Fig. 7: legend of x-axis is partially cut off*

**Author Response:**

We thank the reviewer for this pointing this out.

**Changes to Manuscript:**

Corrected x-axis.

**Comment 12:**

*S. Figs. 18, 19A, 19B refer to Methods only. Please provide the specific*

*section as well.*

**Author Response:**

We thank the reviewer for this pointing this out.

**Changes to Manuscript:**

Added references to specific Methods sections to the captions of these figures.

**Comment 13:**

*The log(k) − τ plot in S. Fig. 20 indicates a TRAIL dose of 50ng/ml. However, the figure caption and the main text on p. 10 only mention a 25ng/ml TRAIL dose.*

**Author Response:**

We thank the reviewer for this pointing this out.

**Changes to Manuscript:**

Replaced the figure with the 25ng/ml TRAIL dose from Roux et al.

**Comment 14:**

*The following sentences do not seem to be entirely correct:*

*•line 267: We centered our the prior for...*

*• line 273: ...and shown Figures 3B.*

*• line 328: ...follow a well-establish and simple...*

*• line 380: ...shows the predicted probabilities of the ordinal categories (as a*

*function predicted cellular tBID content);*

*• line 418: To explore a practical application of the data-driven measurement*

*model paradigm. We...*

*• line 427: ...are indirect semi-quantitative measurements since lack a clear*

*mapping between...*

*• line 490: ...were sensitive to we encode assumptions in the measurement*

*model,...*

*• line 830: To in encode empirical ordering constraints on the...*

*• line 982: ...Guassian process...*

*• line 984: ...Guassian process...*

**Author Response:**

We thank the reviewer for this pointing these out.

**Changes to Manuscript:**

Edited the typos in these lines.

**Comment 15:**

*Comment 17: In response to this comment the authors added on p. 14, line 659, To represent the irreversible progression toward an apoptotic cell fate (and monotonic accumulation of apoptosis cell fate markers, tBID and cPARP), each ordinal constraint function is combined, using the sequential model... And, on the same page, line 662, The cumulative, and adjacent-category models may also be appropriate in other contexts. I find this response somewhat too thin. Especially the second sentence on the cumulative and adjacent-category models does not add much in my view and, as it stands, should be removed. Also, is the irreversible progression the reason why the authors chose the sequential model over the others? Or is it the monotonic accumulation of the markers? Please*

*clarify and motivate/explain briefly the choice of the sequential model. Could the other two models used here? Why or why not (briefly)?*

**Author Response:**

We thank the reviewer for these questions as it prompted us to further into the ordinal models that can potentially apply to our study. Our model uses the Mord [1] package, by Pedregosa et al. 2017 to predict the probability of ordinal class membership. A more detailed reading of their code <https://github.com/fabianp/mord> and supporting dissertation [2] confirms to us that the ordinal models provided in Mord are more accurately described as cumulative. The cumulative model is similar and a generally more appropriate model for ordinal data that arises from categorization of an underlying continuous latent variable. Gerhard Tutz gives more details about the Cumulative, Sequential and Adjacent Category models. They describe how the models differ in interpretation and provide some circumstances where each model may be justified. They also show that these models often produced very comparable estimates and goodness-of-fit [3]. We revised this section with a more accurate description of our approach and we have added a reference to the work by Gerhard Tutz.

[1] Pedregosa, Fabian, Francis Bach, and Alexandre Gramfort. "On the consistency of ordinal regression methods." *The Journal of Machine Learning Research* 18.1 (2017): 1769-1803.

[2] Pedregosa-Izquierdo, Fabian. *Feature extraction and supervised learning on fMRI: from practice to theory*. Diss. Université Pierre et Marie Curie-Paris VI, 2015.

[3] Tutz, Gerhard. "Ordinal regression: A review and a taxonomy of models." *Wiley Interdisciplinary Reviews: Computational Statistics* 14.2 (2022): e1545.

**Changes to Manuscript:**

Significantly revised the paragraph that references the cumulative model (Starting Line 669). We added Equation 15, which describes the ordinal model we used. We also added a reference to Tutz, Gerhard (2022) which gives more information to support the readers’ choice ordinal models.
